# A flexible electromagnetic wave-electricity harvester

**DOI:** 10.1038/s41467-021-21103-9

**Published:** 2021-02-05

**Authors:** Hualiang Lv, Zhihong Yang, Bo Liu, Guanglei Wu, Zhichao Lou, Ben Fei, Renbing Wu

**Affiliations:** 1 Department of Materials Science, Fudan University, Shanghai, 200433 China; 2 College of Materials Science and Technology, Nanjing University of Aeronautics and Astronautics, Nanjing, 210016 China; 3 College of Mechanical and Vehicle Engineering, Hunan University, Changsha, 410082 Hunan China; 4 Institute of Materials for Energy and Environment, State Key Laboratory of Bio-fibers and Eco-textiles, College of Materials Science and Engineering, Qingdao University, Qingdao, 266071 China; 5 College of Materials Sciences and Engineering, Nanjing Forestry University, Nanjing, 210037 China

**Keywords:** Devices for energy harvesting, Electronic devices, Nanoscale materials

## Abstract

Developing an ultimate electromagnetic (EM)-absorbing material that can not only dissipate EM energy but also convert the generated heat into electricity is highly desired but remains a significant challenge. Here, we report a hybrid Sn@C composite with a biological cell-like splitting ability to address this challenge. The composite consisting of Sn nanoparticles embedded within porous carbon would split under a cycled annealing treatment, leading to more dispersed nanoparticles with an ultrasmall size. Benefiting from an electron-transmitting but a phonon-blocking structure created by the splitting behavior, an EM wave-electricity device constructed by the optimum Sn@C composite could achieve an efficiency of EM to heat at widely used frequency region and a maximum thermoelectric figure of merit of 0.62 at 473 K, as well as a constant output voltage and power under the condition of microwave radiation. This work provides a promising solution for solving EM interference with self-powered EM devices.

## Abstract (web summary)

Materials that can harvest electromagnetic (EM) waves and harness the resulting energy would have many applications. Here, the authors present a hybrid composite that produces thermoelectricity from the heating in the EM absorption under microwave radiation.

## Introduction

Continued advances in science and technology enable electronic devices and systems are being made smaller, operated faster, and gotten smarter^1–3^. The miniaturization, high speed, integration, and intellectualization of devices and chips are at the expense of a substantially increase the electromagnetic interference (EMI) and heat generation, which detrimentally affect their performance, reliability, as well as the surrounding environment^4–6^. Currently, most studies concentrate on the solving the EMI alone and a variety of EMI-shielding materials via the reflection or absorption of EM waves have been explored, while the overheating problem of electronic devices was largely ignored^7^. In addition, the absorption of EM waves through the dielectric and/or magnetic dipoles of a material that interact with the radiation and the internal reflection would convert EM energy to thermal energy, further resulting in extra burden of heat generation temperature rising^8^. Recently, the development of a multicomponent heterostructure with good heat dissipation ability and flexibility together with efficient EMI-shielding capability has been regarded as a solution^9^. Nevertheless, exploring advanced heterostructures for small devices and components that can use such wasted thermal energy instead of just dissipating and even realize a self-powering function is more desirable but remains grand challenging.

Thermoelectric (TE) energy involves a direct conversion between thermal and electrical energy with no moving parts and thus exhibits a great potential for the utilization and recycling of waste heat^10,11^. In particular, flexible TE materials are receiving increased attention since they can not only provide a close contact between a heat source surface and arbitrary shapes to achieve an improved energy conversion but also be easily integrated into electronic components, wearable devices, and bioelectronics^12,13^. TE performance is evaluated by the figure of merit, ZT = *σS*^*2*^*T/κ*_T_, where *S, σ, T* and *κ*_T_ represent the Seebeck coefficient, electrical conductivity, absolute temperature, and thermal conductivity, respectively^14^. Inorganic-organic hybrids are a promising candidate for flexible TE materials at appropriate temperature since the TE power factor (defined as PF = *σS*^2^*T*) may reach that of the inorganic materials while the organic components possess lower thermal conductivity and greater mechanical flexibility^15^. For example, a hybrid flexible superlattice comprising alternating layered TiS_2_ monolayers and organic cations^16^, a poly (3,4-ehtylenedioxythiophene)/Bi_2_Te_3_^17^, and Ag_2_Se film on a nylon membrane^18^ could achieve ZT values of 0.28 at 373 K, 0.58 at room temperature, and 0.6 at 300 K, respectively. In this regard, the effective integration of EMI-shielding and TE functions into one flexible material may provide the most promising way against EMI and avoid overheating of electronics. Nevertheless, developing organic–inorganic hybrids with both a strong EMI-shielding capability and a high ZT value is still a formidable challenge since the coupled relations among the ability for the conversion from EM to heats (defined as EM dissipation factor *η*), *S*, *σ*, and *κ* makes the simultaneous improvement of *η* and ZT a nontrivial task^19^.

Herein, we report and demonstrate a combination of architecture design and composite regulation to address this challenge. A designed structure was constructed by selectively depositing organic parylene-c (PL) layer and Au patterns onto the front and the back side of Sn@C film. The front PL layer, intermediate film, and back patterns were functioned as a wave-transmitter, energy harvester, and wave-blocker, respectively. The key energy harvester was made by monodispersed Sn nanocrystals embedded within a porous carbon layer (Sn@C) that originated from the carbon thermal reduction of the SnO_2_@polysaccharide biopolymer (SnO_2_@PB) precursor and exhibited an interesting biological cell-like splitting ability.

## Results

### Splitting of the Sn nanoparticles  with a phase conversion and size reduction

**Fig. 1:**
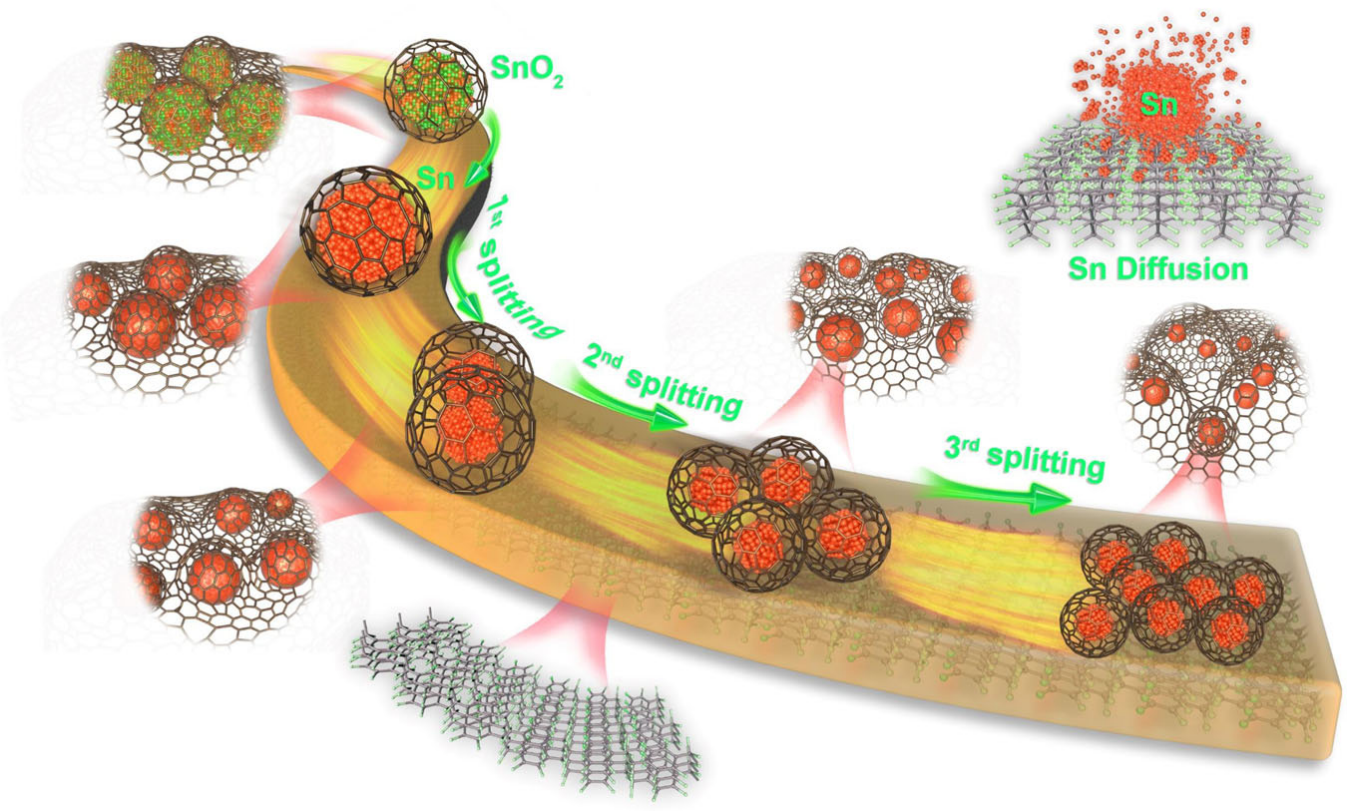
Illustration the procedure of Sn splitting in the carbon matrix. As a result of the cycled annealing treatment, the inserted metallic Sn nanoparticles exhibited a biological cell-like behavior and split into nanoparticles with smaller sizes. Additionally, the phase transitions from α-Sn to β-Sn and from amorphous carbon to graphitic carbon occurred.

**Fig. 2:**
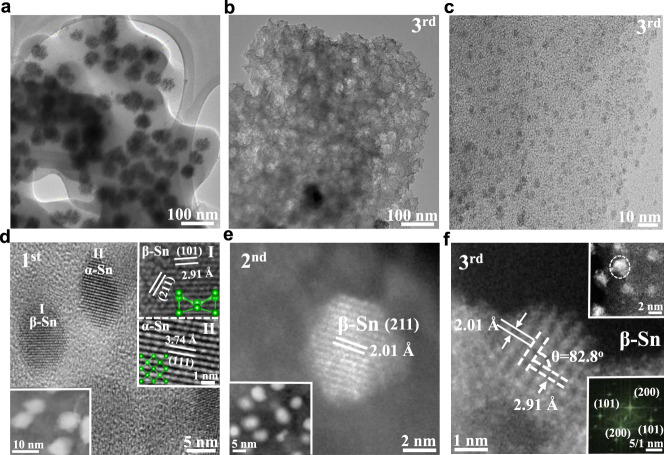
TEM images. **a** TEM image of the SnO_2_ nanoparticles embedded within the carbon matrix (amorphous carbon flake); **b** low- and **c** high-magnified TEM images of the representative third Sn@C. **d** HRTEM image of first Sn@C, the lower left inset showing the HADDF-STEM image and the upper right inset showing the enlarged HRTEM images collected from area I and II. **e** HAADF-STEM image of second Sn@C, the lower left inset showing the low-magnification HAADF-STEM image. **f** HAADF-STEM image of third Sn@C, the upper right inset showing the low-magnification HAADF-STEM image and the lower right inset presenting the fast Fourier transformed (FFT) pattern.

As illustrated in Fig. 1, after cycled annealing treatment, the embedded Sn nanocrystals exhibited a biological cell-like behavior and would split into smaller nanoparticles (NPs) and diffuse into the interior of carbon matrix, leading to a suppressed electron scattering but phonon-blocking structure with an optimized *σ* and *κ*_T_. X-ray diffraction (XRD, Supplementary Fig. 1) pattern analysis and transmission electron microscopy (TEM) images revealed that the initial precursor consisted of embedded SnO_2_ cores with the average sizes of ~37 nm and amorphous carbon flake (Fig. 2a). After annealing treatment cycling, the SnO_2_ could be reduced to metallic Sn and then it got smaller and smaller due to the cell-like division. Consequently, the original cores located by SnO_2_ in carbon flake disappeared, resulting in a mesoporous structure, as evidenced by the Brunauer–Emmett–Teller measurements (Supplementary Fig. 2). The occurrence of splitting and diffusion might be due to the thermal-driven of Sn NPs with low melt points. The formation mechanism of the mesoporous structure and core-evolution were also revealed by an in situ time-dependent of TEM characterization (Supplementary Fig. 3). Figure 2b, c showed representative low-resolution TEM images of Sn@C flake composites obtained after third annealing treatment cycle. It can be observed that numerous nanoparticles were well-dispersed within the carbon and had an average size of ~3.0 nm, significantly smaller than those of the composites obtained after first (~10.0 nm) and second (~7.0 nm) cycled annealing treatment (Fig. 2d, e insets and Supplementary Fig. 4). Nevertheless, as annealing treatment continued to the fourth cycle, the sizes of Sn nanoparticles began to become uneven (ranging from 2 to 20 nm), possibly be due to the unsynchronized re-aggregation and splitting behavior (Supplementary Fig. 5). In addition to the size variation, the cycled annealing treatment had an important effect on the structural phase transition of the Sn. The Sn NPs produced by the first annealing treatment were mainly mixed α- and β-Sn, where a lattice fringe with a spacing of 3.74 Å was ascribed to the (111) crystal plane of α-Sn and the lattice fringes of 2.01 and 2.91 Å were assigned to the (211) and (200) crystal planes of β-Sn, respectively (Fig. 2d and Supplementary Fig. 6). The aberration-corrected high-angle annular dark-field scanning TEM images in Fig. 2e, f and the fast Fourier transform pattern (Fig. 2f inset) confirmed that only the cubic phase of Sn with a resolved 0.201 and 0.291 nm lattice fringes existed in the Sn nanoparticles obtained by the second and the third annealing treatments. The structural transition from mixed *α* and *β* to pure *β* phase in the Sn nanoparticles is believed to be favorable to the optimization of the key parameters *σ* and *κ*_T_.

### Coupled EM wave-thermal-electricity performance

**Fig. 3:**
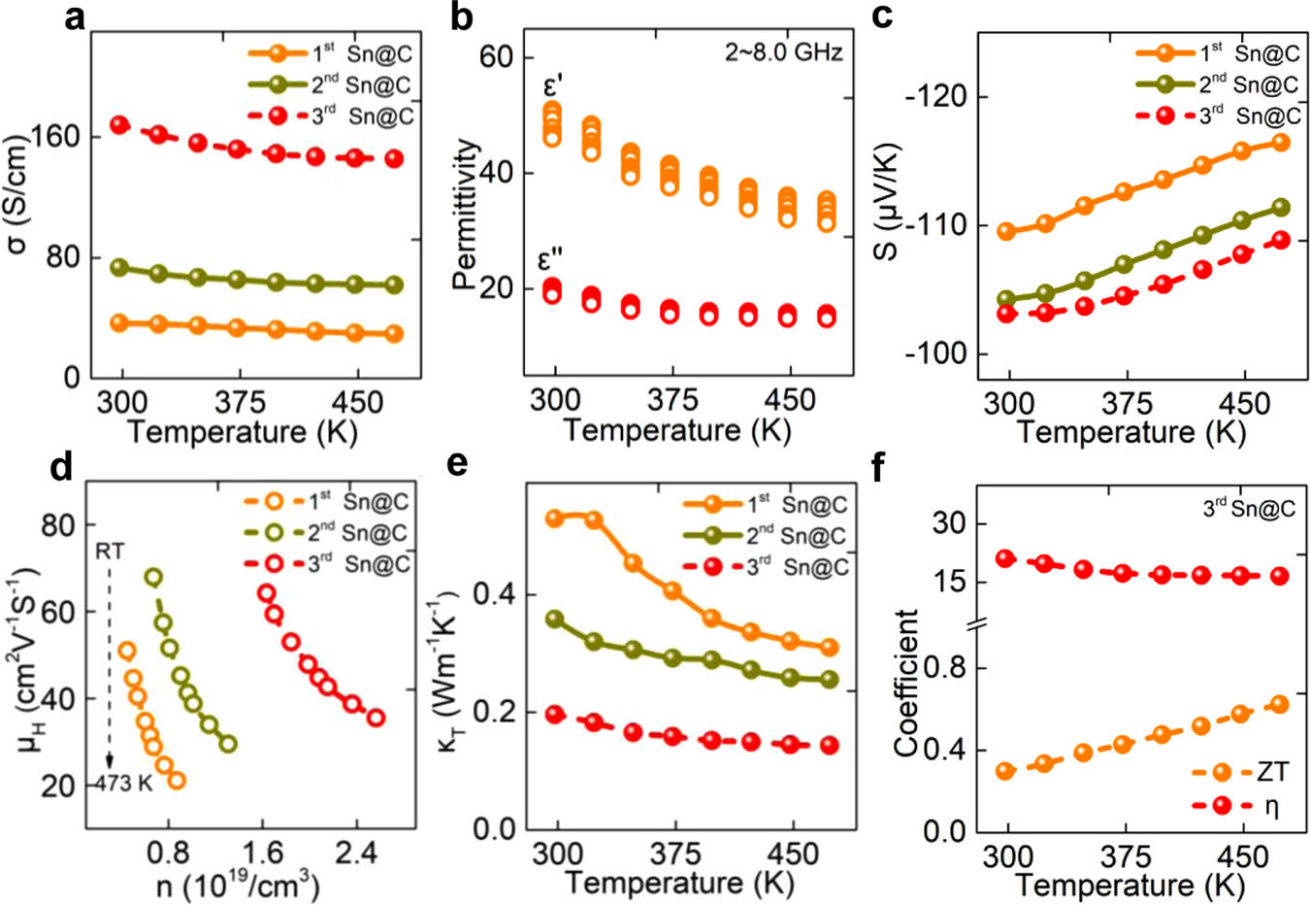
Analysis of coupled EM wave-to-electricity performance. **a** In-plane conductivity (*σ*); **b** the real and the imaginary part of permittivity; **c** Seebeck coefficient; **d** Hall carrier concentration (*n*) as a function of the mobility (*μ*); **e** total thermal conductivity (*κ*_T_); **f** coupled EM wave-thermal-electricity performance from 298~473 K (applied frequency region: 2~8.0 GHz).

The temperature-dependent electrical conductivity (*σ*), intrinsic permittivity and Seebeck (*S*) of Sn@C samples made with cycled annealing treatment were plotted in Fig. 3a–c. The electrical conductivities of all samples decreased with increasing temperature (Fig. 3a), displaying a degenerated semiconductor transport behavior. Specifically, the *σ* of the third Sn@C composite ranged from 168 to 146 S/cm at the measured temperature region, nearly five times higher than that of the first Sn@C composite. The observed increase in *σ* of these Sn@C composites was attributed to the structural phase transition from α-Sn to β-Sn, reduced grain boundaries and varied electronic structure of the carbon flake (Supplementary Note 1). Figure 3b and Supplementary Fig. 7 plotted the temperature-dependent of the real and the imaginary part of permittivity (*ε*′, *ε*″) measured from 298 to 473 K. As the temperature rising, both of *ε*′ and *ε*″ displayed a decreasing tendency, showing the similar tendency to that of *σ*, which can be qualitatively explained by the free electron theory (Supplementary Note 2 and Supplementary Fig. 8)^20^. The *S* values as a function of temperatures were compared in Fig. 3c. Notably, the negative *S* values throughout the tested temperature range indicated that they were *n*-type nature and the electrons were the predominant carriers for all samples^21^. From 298 to 473 K, the *S* values were estimated to be −109 to −116, −105 to −111, and −103 to −108 μV/K for the first, second, the third Sn@C composites, respectively. For metals or degenerate semiconductors, the lowest absolute Seebeck coefficient (|*S*|) for the third Sn@C composite could be attributed to its largest carrier concentration (*n*), as demonstrated by the mobility (*μ*_H_) as a function of hall *n* (*μ*_H_ = *σ/en*, where *e* is the electron charge) over the entirely measured temperature region (Fig. 3d)^22,23^. A larger effective mass (m*) and a smaller *n* contributed to a high (|*S*|) value. Compared with the mobilities of the first and the second Sn@C samples, the mobility *μ*_H_ of the third Sn@C composite was the largest (64.2~35.5 cm^2^ V^−1^ S^−1^), indicating a decreased m*^24,25^. Meanwhile, the *n* increased apparently from 0.45~0.87×10^19^ cm^−3^ for the first Sn@C to the 1.6~2.6×10^19^ cm^−3^ for the third Sn@C. The smallest m* and the largest *n* means that the |*S* | coefficient of the third Sn@C is the lowest in comparison with those of the first, the second samples. The temperature-dependent total thermal conductivity (*κ*_T_) for these samples was presented in Fig. 3e. The *κ*_T_ for the first, the second Sn@C and the third Sn@C composites continuously decreased with increasing temperature from 298 to 473 K due to enhanced phonon-phonon scattering and suppressed electron scattering. The lowest *κ*_T_ value of 0.2~0.15 Wm^−1^ K^−1^ was observed for the third Sn@C composite, suggesting that the presence of structural transition from α to β-Sn and the high dispersion of ultrasmall Sn NPs within carbon matrix played a significant role in the reduction of *κ*_T_.

The utilization of unwanted EM wave to generate electricity involves two procedures: (i) dissipating ambient EM wave to heat; (ii) converting wasted heat to electricity. The dissipation ability can be evaluated according to Eq. (1)^26^:

1 $${\mathrm{W}} = D \cdot E^2 \cdot f \cdot \eta$$

where *D* is a coefficient associated with the volume of an absorber, *E* and *f* represent the power and frequency of EM wave, respectively. *η* is EM dissipation factor, which can be calculated by Eq. (2)^27^:

2 $$\eta \,=\, \varepsilon _r \cdot \tan \delta _E = \varepsilon _r \cdot \varepsilon ^{\prime\prime} /\varepsilon ^{\prime}$$

where *ε*_*r*_ is the relative complex permittivity and tan *δ*_*E*_ is the dielectric tangent loss, equaling to the ratio of *ε*″/*ε*′ (*ε*′and *ε*″ are real and imaginary part of permittivity values, respectively).

To maximize the utilization efficiency of EM energy, the *η* and ZT values of the as-synthesized Sn@C composites should be as large as possible. As shown in Fig. 3f and supplementary Fig. 9, the third Sn@C sample not only exhibited a strong EM dissipation factor over 16.6 ranging from 2.0 to 8.0 GHz (mainly working frequency for intelligent electronics such as 4/5 G wireless, WIFI, and Bluetooth) but also could achieve a ZT value of 0.62 at 473 K, meaning a possibility of coupled EM wave-thermal-electricity. This *η* is superior to most reported EM shielding or absorbing materials as well as TE materials (Supplementary Figs. 10 and 11). The ZT value is also comparable to those of commonly organic or inorganic TE materials (Supplementary Table 1). By contrast, both the first and the second Sn@C samples possessed a very limited ZT values between 0.05~0.15 at 473 K, indicating a poorly coupled performance even if they had a comparable *η* value over 10.

### Insights into the exceptional coupled performance

The third Sn@C with higher conductivity value would lead to a large *ε*_*r*_ value based on the free electron theory^28^. Meanwhile, the as-fabricated core-shell structure consisting of graphitized carbon shell and well-dispersed Sn NPs core were also favorable to the tangent loss value, which could be explained by equivalent circuit theory^29^. Therefore, a desirable *η* was obtained for third Sn@C composite.

**Fig. 4:**
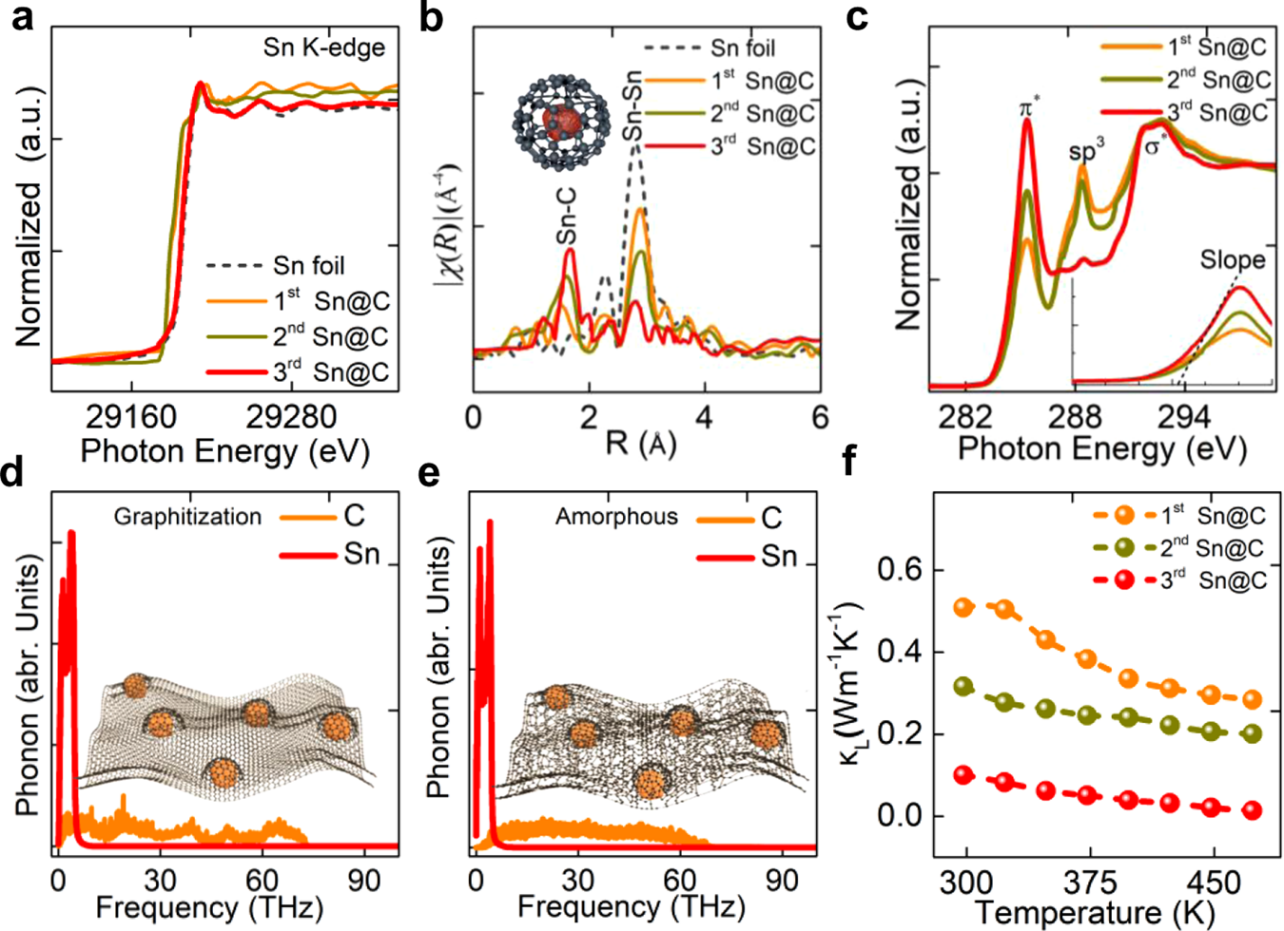
Mechanism for the coupled performance. **a**, **b** EXAFS of Sn *K*-edge and *κ*^3^-weighted *χ*(R) function of EXAFS spectra; **c** XANES C 1*s* edge spectra for the sample obtained with different annealing treatment cycling; **d**, **e** calculated phonon spectra for the Sn-graphitized/amorphous carbon matrix, and **f** temperature dependence of lattice thermal conductivity (*κ*_L_).

The reason for the high ZT value of the third Sn@C composite could be ascribed to the synergistic effect of the ultrasmall β-Sn NPs and carbon matrix. As demonstrated by a comparison of the experimental results, the sample made with pure carbon matrix (Sn NPs were removed by HCl) had a higher *κ*_T_, significantly decreased *σ* and |*S*|, and thus a poor ZT value (less than 0.01) (Supplementary Fig. 12). The extended X-ray absorption fine structure (EXAFS) technique was further employed to elucidate the structural advantage of Sn@C composites. An apparent variation could be observed in the EXAFS spectra of the Sn K-edge for first, second and third Sn@C composites (Fig. 4a). The EXAFS spectrum in R space further revealed that with increasing the annealing treatment time, the Sn-Sn coordination at ~2.8 Å were gradually weakened, whereas the Sn-C coordination (~1.6 Å) increased in strength (Fig. 4b), confirming that the splitting of the Sn changed its local coordination geometry and increased the quantity of nanoparticles^30,31^. On the other hand, the increased numbers of Sn NPs with decreased sizes also affected the chemical environments around the C, as investigated by the X-ray absorption near-edge structure (XANES) technique. The observed XANES spectra of C in Fig. 4c displayed four peaks, centering at 285.3, 288.3, 291.7, and 292.9 eV, respectively. The former two peaks corresponded to 1*s*→π* and π* from non-graphitized atom, respectively, whereas the latter two peaks could be attributed to 1*s*→σ*^32^. The unoccupied π* and σ* states indicated that the carbon was mainly in a *sp*^2^-like hexagonal structure, despite the presence of a disordered part^33^. Nevertheless, as the size of Sn nanoparticles decreased, the relative intensity of 1*s*→π* peaks were gradually enhanced, and the disordered intensity were remarkably weakened, indicating that the carbon matrix began to be graphitization^34,35^.

In addition to the XANES spectra, the Raman and X-ray photoelectron spectra results also demonstrated that an increase in annealing treatment cycle times would be favorable to the graphitization level (Supplementary Note 3 and Supplementary Figs. 13 and 14). The increased graphitization level was believed to improve the electrical conductivity. More importantly, the thermal conductivity could be drastically reduced by the phonon coupling (PC) between the Sn nanoparticles and graphitized carbon atoms. To provide an insight into the contribution of coupling effect on the thermal conductivity, we conducted the non-equilibrium molecular dynamics simulations to understand the PC mechanism. The details of the interatomic potential parameters and computation methodology could be found in Supplementary Note 4. In the simulations, we observed that the frequency of the phonons of Sn atoms mainly fell in the range from 0 to 10 THz, whereas the frequency of the phonons of C atoms for both amorphous and crystalline graphite ranged from 0 to 70 THz (Fig. 4d, e). Compared with amorphous counterpart, the graphitized carbon could allow more phonons located from 0 to 10 THz and boost the coupling intensity. Quantitatively, the PC can be calculated by the overlap of the phonon spectra as following^36^:

3 $${\mathrm{PC = }}\left( {{\int} {\sqrt {{\mathrm{P}}_1\left( \omega \right){\mathrm{P}}_2\left( \omega \right)} {\mathrm{d}}\omega } } \right)^2/\left( {{\int} {{\mathrm{P}}_1} \left( \omega \right){\mathrm{d}}\omega {\int} {{\mathrm{P}}_2\left( \omega \right){\mathrm{d}}\omega } } \right)$$

where P_1_(ω) and P_2_(ω) are the phonon spectra for the C and Sn atoms, respectively. The values of the PC were obtained as 0.018 and 0.114 for the amorphous and graphitized carbon, respectively. The stronger PC intensity sharply boosted the phonon scattering between Sn NPs and surrounding C atoms, which resulted in a high thermal resistance and make great contribution to an ultralow lattice thermal conductivity (*κ*_L_, $\kappa _{\mathrm{L}} = \kappa _{\mathrm{T}} - \kappa _{\mathrm{e}}$,where *κ*_e_ refers to the electron thermal), according to Fig. 4f^37^. The detailed calculation method of *κ*_e_ can be found in Supplementary Note 5. Moreover, the effect of the size and number of Sn NPs on the thermal conductivity was also studied by theoretical simulation (Supplementary Fig. 15). Apart from *κ*_L,_ the Sn NPs embedded within the carbon matrix may also be benefited to a low *κ*_e_, according to Supplementary Figs. 16 and 17. Owing to the smallest sum of *κ*_e_ and *κ*_L_, the third Sn@C device had an ultralow *κ*_T,_ as compared to that for first and second Sn@C, thus played a vital role on higher ZT value.

### Construction of a flexible EM-electricity harvester

**Fig. 5:**
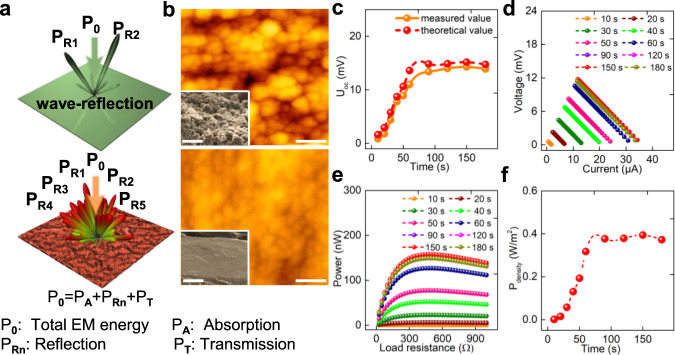
The as-prepared EM wave-electricity harvester. **a** Schematic illustration of EM wave reflection on the PL and Sn@C interlayer; **b** atomic force microscope (AFM) images of Sn@C before and after depositing PL layer (scale bar 50 μm); the inset showing the corresponding surface condition observed by FESEM (scale bar 10 μm). **c** Time-dependent of open-circuit voltage, **d** output voltage-current, **e** output power and **f** power density for the EM wave-electricity harvester. Note that the changed voltage and current could be recorded by tuning the load resistance from 0~1000 Ω.

To demonstrate an exceptional coupled performance, four legs of the third Sn@C composite film and the flexible polydimethylsiloxane (PDMS) as an insulated substrate were employed to construct a device (Supplementary Fig. 18). Before construction, a PL layer with 0.5 cm in length was selectively deposited onto the front side of the Sn@C leg via a chemical vapor deposition (CVD), while Au-rectangle patterns were thermal-evaporated onto its back side using a specific mask (Supplementary Fig. 19). The selectively deposited PL layer is conducive to improving the EM dissipation factor and creating a large temperature gradient. Specifically, the PL layer would reduce the direct wave reflection due to its excellent wave-transmission ability and simultaneous reduction in wave-scattering after achieving a smooth surface (Fig. 5a). In this regard, more EM waves could enter the interior of the device and then dissipated by the Sn@C film. The effect of the deposited PL thickness on the root mean square (RMS) and the corresponding EM-harvesting coefficient was also investigated. As shown in Supplementary Figs. 20 and 21 and Supplementary Note 6, the optimal PL thickness was fixed to be 460 nm as this condition could enable the lowest RMS (~49 nm), much smaller than that of original Sn@C layer (~274 nm). Accordingly, the Sn@C film with PL layer had a smoother surface (Fig. 5b). Owing to the synergistic effect of excellent wave-transmission and smooth surface, the deposited PL layer allowed more incomings of EM waves for subsequent harvesting by Sn@C leg and resulted in an improved *η*. For the unharvested EM waves, they would be reflected by the deposited Au-rectangular pattern and the second or/and even multiples harvested Sn@C layer, thus further improving the EM wave-harvesting efficiency. The effect of structural design on the EM-harvesting ability was also confirmed by the EM simulation results (Supplementary Note 7). In addition to an enhanced *η* value, the deposited PL layer possessed an excellent thermal-insulation performance (only ~0.07 W m^−1^ K^−1^), thus would avoid the quick thermal diffusion in deposited areas and further make contribution to maintaining the temperature gradient. In addition, the thermal stability of deposited areas exhibited a satisfied heat-resistance performance, as discussed in Supplementary Fig. 22 and Supplementary Note 8.

The temperature difference (Δ*T*) was realized by EM radiating the Sn@C leg for 0~180 s using a homemade microwave emitting cavity. The magnetron (~100 W) as the core part of microwave emitting cavity could generate a ~2.45 GHz of EM waves (Supplementary Fig. 23). The Δ*T* as a function of radiation times were recorded via an infrared thermometer (Supplementary Note 9). The maximum Δ*T* could reach 36 K after radiating 150 s (Supplementary Fig. 24). The open-circuit voltage (*U*_oc_), output power and current-voltage of EM-electricity harvester were shown in Fig. 5c–f. The measured *U*_oc_ exhibited a remarkable enhancement as radiation time increasing to 90 s and then gradually maintained a constant value (Fig. 5c). The maximum *U*_oc_ value reached ~14.3 mV after radiating 150 s, which was ascribed to the maximum Δ*T*. Commonly, the theoretical *U*_oc_ value could be estimated by the equation $U{_{\mathrm{oc}}} = {\Delta}T \times N \times \overline {\left| S \right|}$, where *N* is the number of leg, $\overline {\left| S \right|}$ is the average absolute Seebeck coefficient, ~105.4 μV/K. The measured *U*_oc_ values exhibited slightly lower than that of theoretical value, which was due to fact that the time delay (~15 s) from completing radiation to voltage-characterization would narrow the Δ*T* value. Figure 5d plotted the output current vs. voltage curves for the EM device after radiating various times and altered load resistance ranging from 0~1000 Ω. We observed that the maximum output voltage was ~12 mV. Based on *U*–*I* curve, the output power (*P*_out_) could be calculated by the follow expressions^38^

4 $$P_{{\mathrm{out}}} = I^2R_{{\mathrm{ex}}} = \left( {\frac{{U_{{\mathrm{oc}}}}}{{R_{{\mathrm{in}}} + R_{{\mathrm{ex}}}}}} \right)^2R_{{\mathrm{ex}}}$$

5 $$V_{{\mathrm{out}}} = I \cdot R_{{\mathrm{ex}}}$$

where *I* represents the output current, *R*_ex_ and *R*_in_ are the load resistance and internal resistance of leg, respectively, *V*_out_ refers to the output voltage. When the *R*_ex_ equals to *R*_in_, *P*_out_ reached a maximum value. As plotted in Fig. 5e, a projected maximum *P*_out_ of ~157 nW could be achieved when radiating the EM device for 150 s and tuning the load resistance to 675 Ω. The maximum *P*_out_ was competitive with the those of state-of-art organic/inorganic TE nanogenerator, as listed in Supplementary Table 2. Apart from output power, the power density could be determined by $P_{{\mathrm{density}}} = \frac{{P_{\max }}}{{N \cdot A}}$, where *A* presents the crossed area of the leg. Similarly, after 150 s of radiation, the EM device reached the maximum power density of 0.394 W/m^2^ (Fig. 5e, f).

To investigate the flexibility of EM nanogenerator, a bending test was conducted using the original resistance (*R*_o_) as a reference (Supplementary Note 10). Before the test, the EM device was folded for 600 times and then attached to the surface of an insulated tube (diameter ~10 cm) with different contact degrees. Supplementary Fig. 25 showed the changes of electrical resistance (*R*_s_) as a function of the bending angle. Although the ratio of *R*_s_*/R*_o_ slightly changed at varied bending degree, it was always less than 1.07, demonstrating the excellent flexibility caused by the organic–inorganic hybrid component. This result firmly indicated that such an EM device could be attached on any curved surface to achieve an excellent coupled EM-to-electrically ability, which was significantly different from the well-investigated TE materials such as SnSe, Ag_2_Te, and Bi_2_Te_3_ or conventional EM dissipation materials-based devices.

## Discussion

In summary, we have demonstrated a flexible EM device constructed by a cell splitting-like Sn@C composite film with a selective deposition of organic PL layer, enabling an excellent electromagnetic wave-heat-electricity ability. Owing to a suppressed electron scattering but phonon-blocking structure caused by the split of Sn as well as its phase transition within the carbon matrix via a cycled annealing treatment, the EM device exhibited an exceptionally coupled EM wave-heat-electricity performance with a maximum output power ~157 nW and output voltage ~12 mV under EM wave radiation ~15 s. This work may provide a solution to solve EMI and open opportunities for harvesting waste EM energy.

## Methods

### Synthesis of SnO_2_@PB

The SnO_2_@PB microflakes were prepared by a facile hydrothermal route^9^. In details, D-glucose (9.0 g) and Na_2_SnO_3_ (0.325 g) were co-dispersed into 40 mL distilled water under mechanical stirring for 30 min. Afterward, the above solution was transferred into an autoclave and maintained at 180 °C for 4 h. After cooled to room temperature, the SnO_2_@PB could be collected by centrifugation, washed several times with absolution ethanol, and dried under vacuum at 60 °C.

### Preparation of Sn@C composites

Sn@C microflakes could be achived by a thermal reduction of SnO_2_@PB at 900 °C for 2 h under a flowing mixed gas (first annealing treatment). To get Sn nanoparticles with smaller size, the as-prepared Sn@C composites were treated under cycled annealing and the detailed experiment conditions can be referred in Supplementary Table 3. To ensure a homogeneous spiliting speed, the rates of heating gradually increased as increasing the cycled treatment times.

### Fabrication of a flexibile EM-electricity harvester

To fabricate harvestor, a thin film composed by Sn@C composites (thickness ~0.02 mm) was made by a spin-casting method on a F-containng glass substrate. Then, one side of the film was employed to selectively deposite the orgnaic PL layer using a CVD. The CVD process involved four-temperature stages, i.e., 120, 130, 140, and 150 °C, corresponding times fixed as 20, 20, 40, and 40 min. In addition, the temperature of the chamber for polymerization was set to 650 °C. The PL monomer was placed in another chamber and corresponding tempreature was seted as 120 °C. The thickness of the PL was determined by the initial amount of PL monomers. After peeling off from the F-containing glass, the film comprising of two layers can be obtained and then attached on metal blackplane for the subsequent thermal evaporation. In this case, the patterned Au can be deposited on another side of above film. A specific mask was used to control the length, width and thickness (in our case, thickness ~50 nm). Afterwards, the top layer (orginal parlyelene layer) was attacted on a glass substrate, another parylene layer was grew on another side (containing Au). The as-fabricated Sn@C film was cut into four legs (25 mm × 5 mm, and the legs were pasted on the flexible PDMS substrate (thickness ~30 μm) with the interval of two legs ~5 mm. After that, each leg was connected in series with Au paste as conductive connection to measure the power output capabilty.

### Characterizations

The phase identification of the samples was recorded using the powder XRD patterns (Bruker D8 ADVANCE X-ray diffractometer) with Cu Kα radiation (*λ* = 0.15406 nm). TEM (JOEL JEM 2100 F) was employed to investigate the particle size and morphology of the prepared samples. The Synchrotron XRD (XAS) was employed out at beamline 33BM-C of XOR Division at Advanced Photon Sources. The graphitization levels of carbon matrix were recorded in the Raman spectrum (Jobin Yvon HR 800 confocal Raman system). The permittivity parameter was conducted on an Agilent PNA N5224A vector network analyzer (details see Supplementary estimation of EM-harvesting performance, based on the transmission-line theory).

## Supplementary information

Supplementary Information

Peer Review File

## Data Availability

The data that support the findings of this study are available from the corresponding author upon reasonable request.
