# Supplementary material for: A flexible electromagnetic wave-electricity harvester
Source: Nat Commun. 2021 Feb 5;12:834. doi: 10.1038/s41467-021-21103-9 (PMC7864982; doi:10.1038/s41467-021-21103-9)
Supplement: Supplementary file 1 — Supplementary Information [file 41467_2021_21103_MOESM1_ESM.pdf]

Supplementary materials

A flexible electromagnetic wave-electricity harvester

Supplementary Methods

Synthesis of Bi_2Te_3

Bi_2Te_3 was made by a facile hyderothemal route[1]. Typically, 0.97 g of bismuth nitrate pentahydrate ($Bi(NO_3)_3$) and 1.2 g of potassium hydroxide (KOH) were co-dissolved in 60 mL ethylene glycol and then stirring for 30 min. Afterwards, 0.38 g of Te and 4 g of 1-Butyl-3-methylimidazolium bromide ($C_8H_{15}BrN_2$) powders were added into the above solution and then stirred vigorously for another 2 h. The solution was then transferred into an autoclave and maintained at 160 ^oC for 6 h. The as-synthesized products were collected by a high-speed centrifugation and washed by the distilled water and absolute ethanol for several times. Finally, the obtained samples were dried at 60 ^oC under vacuum oven for 12 h.

Synthesis of 2H-MoS_2

Firstly, 100 mg of $NaMoO_4$ and 100 mg of CH_3CSNH_2 were mixed into 50 mL of distilled water, and then stirred for 20 min. Subsequently, the solution was transferred into an autoclave and kept at 180 ^oC for 14 h. After cooled naturally, 2H-MoS_2 could be obtained.

Synthesis of Ag_2Te nanorods

Ag_2Te nanorods were typically synthesized according to the previous report[2]. Firstly, 1 mL of hydrazine ($N_2H_4 \cdot H_2O$) was added into 10 mL of 0.01 M $AgNO_3$ aqueous solution, containing 0.1 M Na_2EDTA. Then, 0.1 mmol of Te powder was added into above solution. After aging at room temperature for 30 min, the reaction solution was

kept in a water bath at 80 °C for 30 min. Final product was obtained by centrifuge-separating and waterwashing the black precipitate formed in the solution.

Synthesis of LaSrCo-perovskite

LaSrCo-perovskite was prepared by a chemical route. Typically, stoichiometric amounts of preheated La_2O_3 (1.0 mmol), $SrCO_3$ (1.0 mmol), and cobalt oxalates (1.0 mmol) were ground well and then heated at 950~1000 ^oC with intermediate grindings.

Synthesis of CuS

CuS was prepared by a facile hydrothemal procedure. 1 mmol of $Cu(NO_3)_2$, 0.2 mmol of CTAB and 1 mmol of sulfur (S) were mixed together into the ethylene glycol solution (50 mL) and then stirred for 20 mins. The above solution was then transferred into the aucoclave for the hydrothermal reaction. The temperature and reaction time were set as 140 ^oC and 10 h, respectively. After washed with distilled water for 3~5 times, CuS powder could be synthesized.

Synthesis of ZnO nanorods

ZnO nanorods were synthesized by a facile hydrothermal method. In details, 100 mg of zinc acetate ($Zn(Ac)_2$) were dissolved in 50 mL of distilled water with magnetic stirring for 30 min. Afterward, 1.0 mL of ammonia solution was added dropwise into the above solution. The transparent solution was then transferred into an autoclave for the hydrothermal and heated at 160 °C for 8 h. The white precipitate was collected after washing with distilled water 3~5 times.

Synthesis of MFe_2O_4@C (M=Fe, Co)

Before preparing MFe_2O_4@C, MFe_2O_4 nanoparticles (NPs) were made by a simple hydrothermal approch. Typically, 1 mmol $FeCl_2$ (or $Co(Ac)_2$) and 2 mmol $FeCl_3$ were co-mixed into a 50 mL EG solution and then stirred 20 min. The urea (15 mmol) was then added into the above solution and transferred into 100 mL autoclave. The

autoclave was heated at 180 ^{o}C for 12 h. After the temperature cooled to room temperature, the as-obtained precipitates were collected, washed with distilled water and ethanol 3~5 times, and treated at 80 °C for 10 h. To prepare MFe_2O_4@C composite, 0.1 g of as-prepared Fe_3O_4 or $CoFe_2O_4$ powders were dispersed into a solution containing 60 mL distilled water, 60 mL ethanol solution and 0.8 mL $NH_3 \cdot H_2O$ with ultrasonic mixing for 1 h. Afterwards, 0.4 g of resorcinol and 2 mL of formaldehyde were added to the above mixture for polymerization for 24 h. The generated Fe_3O_4@phenolic resin was then heated at 600 °C in a N_2 flow. The heating rate was controlled at 5 °C/min.

Synthesis of Co_xFe_y@C (M=Fe, Co)

To obtain Co_xFe_y@C, the generated Fe_3O_4@phenolic resin was reduced by hydrogen gas at 500 °C.

Synthesis of Fe/MnO_2 and Fe/MWCNTs

Fe/MnO_2 composite was prepared according to the previous liteature[3]. Rod-like MnO_2 was synthesized first by the straightforward liquid phase process. 15 mmol of $MnSO_4$ and 10 mmol of $KMnO_4$ were dissolved in 100 mL distilled water (DI) and stirred for 20 min to obtain a clear solution, which was then transferred to a water bath and held at 80 ^{o}C for 20 h. After cooling to room temperature, the final product was washed with ethanol under centrifugation. The product was vacuum-dried at 50 ^{o}C. Fe/MnO_2 was produced by a thermal decomposition route using $Fe(CO)_5$ as the Fe source. In details, 0.1 g of rod-like MnO_2 was dissolved in a four-necked flask containing 600 mL kerosene under ultrasonic treatment for 20 min. The flask was then fitted with temperature controller, reflux unit, mechanical agitator and a flow tube. $Fe(CO)_5$ was added under nitrogen and then heated for 6 h at 180 ^{o}C. Final, Fe/MnO_2 could be obtained. Fe/MnO_2 was prepared using the similar process.

Synthesis of Fe_3O_4/graphene

Typically, 20 mg of graphene was first added into a flask containing 100 mL of glycol. Followed by an ultrasonic treatment for 30 min, $FeCl_2$ and $FeCl_3$ were co-added to the mixture and dissolved with the assistance of mechanical stirring for 20 min. Subsequently, the mixture solution was transferred to an autoclave and heated at 140 °C for 24 h. After cooled to room temperature, graphene/Fe-glycolate was obtained by centrifugation and washed with distilled water. To convert the metal-glycolate into metal oxide, the as-prepared graphene/Fe-glycolate was heated at 400 °C under N_2 atmosphere for 2 h with a slow ramping rate of 5 °C/min.

Synthesis of SnS, SnSe and Ni/NiO

SnS and SnSe and Ni/NiO were prepared according to the previsous methods[4-6].

Characterization

The phase identification of samples was recorded using the powder X-ray diffraction (XRD) patterns (Bruker D8 ADVANCE X-ray diffractometer) with Cu Kα radiation (λ=0.15406 nm). Transmission electron microscope (TEM, JOEL JEM 2100F) was employed to investigate the morphology and microstructure of the as-prepared samples. The information of specific surface area and pore size was identified by the N_2-isothermal adsorption-desorption analyzer (Micromeritics ASAP 2010). The Synchrotron X-ray diffraction (XAS) was carried out at beamline 33BM-C of XOR Division at Advanced Photon Sources (APS). The graphitization levels of carbon matrix were investigated by Raman spectrum (Jobin Yvon HR 800 confocal Raman system, wavenumber: 531 cm^{-1}). The electrical conductivity (σ) of films was measured by four-probe method with a Keithley 4200-SCS electrometer (Keithley, Cleveland, Ohio, USA) at room temperature. The thickness was measured by a profilometer (KLA, Tencor). The surface condition was observed by an atomic force

microscope (AFM, Park NX10). The output power was measured by changing the external load resistance. The corresponding current and voltage were collected using a multimeter (Keithley 6482).

Evaluation of EM harvesting performance

The temperature dependent of permittivity parameters was estimated by using an Agilent VNA (Vector network Analyzer, N5232). The samples were made by mixing 30 vol % products with silicon resin and then pressing them into a toroidal ring with an outer diameter 7.0 mm and inner diameter 3.04 mm. The measured permittivity value included two parts: silicon resin and Sn@C sample. The integrated permittivity value of Sn@C can be estimated by the follow equation[7]:

$$\lg \varepsilon = V_1 \lg \varepsilon_1 + V_2 \lg \varepsilon_2 + V_3 \lg \varepsilon_3 + \cdots + V_n \lg \varepsilon_n \quad (1)$$

where V_1, V_2, V_3 and V_n represent the volume ratios of each component. The permittivity of pure silicon resin could be measured first at elevuated temperature. When the permittivity value of silicon resin is known, we could calculate the permittivity of sample based on equation (1).

Characterization of TE-related parameters

The bending electrical conductivity (σ) of EM harvester was measured by four-probe method with a Keithley 4200-SCS electrometer (Keithley, Cleveland, Ohio, USA). The thickness was measured by a profilometer (KLA, Tencor). The surface condition was observed by an atomic force microscope (AFM, Park NX10). The temperature dependent of conductivity and Seebeck coefficient were recorded using ZEM-3 system (Ulvac-Riko, Japan). Before testing, the sample was hot-pressed into a pellet with ~12 mm in length and ~3 mm in width. The thermal conductivity is calculated based on the equation of $\kappa_T = DC_p\lambda$ (where D was the density, C_p is the specify heat value and λ is the thermal diffusivity. The density was determined by the

Archimede method. The thermal diffusivity was measured on the laser flash diffusivity method by LFA-457. Additionally, the specific heat values were collected on a Pyroceram 9606 system. Hall carrier concentration was measured on a commercial system (PPMS, quantum Design).

Supplementary Note 1. Mechanism for the increased σ

The observed increase in σ is attributed to the phase conversion from α to β-Sn, reduced grain boundaries and varied electronic structure of carbon flake.

i) Phase conversion

Commonly, metallic Sn exists in two phases, i.e. α- and β-Sn. Different phases of Sn showed a significantly difference in physical properties, especially for the σ. α-Sn belongs to a diamond cubic system (a=0.6489 nm). Each cell contains eight Sn atoms, which are composed by the covalent bond. On contrast, β-Sn is a kind of tetragonal system (a=0.5832; c=0.3181), and presents a metallic behavior, owing to the metallic bond. Consequently, β-Sn has a higher σ value than α-Sn. In our case, the existed α-Sn can be totally converted to β-Sn after conducting the 2nd cycled annealed treatment, leading to the increased σ. This is the primary reason why σ of 2nd Sn@C is higher than that of 1st Sn@C.

ii) Reduction of grain boundaries

After conducting the cycled annealing treatment, the sizes of Sn NPs were gradually reduced from ~10 nm to 3 nm, suggesting the reduced grain boundaries. Usually, the reduced boundaries would be benefit to the σ value.

iii) Improved graphitization of carbon matrix

During the cycled annealing treatment, the electronic structure of the carbon matrix would change significantly. According to the Raman spectra (Supplementary Fig. 13 and Fig. 14), the intensity ratios of D to G band were gradually decreased after

extending the cycled time, suggesting the improved graphitization degree. The enhancement of graphitization degree means the improved numbers of graphited carbon atoms and the increased conductivity.

Supplementary Note 2. Mechanism for the varied permittivity

Intrinsinc primitivity of one material reflects the sum of polarization relaxation and conductivity loss. Commonly, dielectric polarization (ε''_p) mainly arises from various the local movement of bound charges and the variation of dipole moment in the alternated EM filed. When polarization relaxation occurs, it can affect both ε' and ε'' values. Accordingly, two typical phenomena can be found, that is, sharply decreased ε' value due to the frequency dispersive effect, and dielectric resonance peak in ε''. Regarding the frequency dispersive, the plots of ε' *versus* ε'' would be a single semicircle, normally denoted as the Cole-Cole semicircle, according to the classic Debye-theory. Specifically, the relative complex permittivity can be drawn as following[8,9]:

$$\varepsilon_r = \varepsilon_\infty + \frac{\varepsilon_s - \varepsilon_\infty}{1 + j2\pi f\tau} = \varepsilon' - j\varepsilon'' \quad (2)$$

Where ε_s, ε_∞, τ are static permittivity, relative dielectric permittivity at high-frequency limit, and polarization relaxation time, respectively. After the separation of real and imaginary parts, the following equations could be obtained:

$$\varepsilon' = \varepsilon_\infty + \frac{\varepsilon_s - \varepsilon_\infty}{1 + (2\pi f)^2\tau^2} \quad (3)$$

$$\varepsilon'' = \frac{2\pi f\tau(\varepsilon_s - \varepsilon_\infty)}{1 + (2\pi f)^2\tau^2} \quad (4)$$

Based on the equation (2) and (3), the ε'-ε'' can be expressed:

$$(\varepsilon' - \varepsilon_\infty)^2 + (\varepsilon'')^2 = (\varepsilon_s - \varepsilon_\infty)^2 \quad (5)$$

According to equation (4), each Cole-Cole semicircle is corresponding to one Debye

relaxation process. Supplementary Fig. 8 reveals that there is no significant Cole-Cole curve for the 1st, 2nd and 3rd Sn@C samples even if the temperature rising. Hence, the influence of polarization relaxation on the permittivity values can be ignored at measured 2-8.0 GHz. In this regard, we can deduce that the conductive loss plays a dominate role on the permittivity. Commonly, the conductive loss intensity has a close correlation with the conductivity (σ), based on the free-electron theory [10]:

$$\varepsilon'' \approx \varepsilon_c'' = \sigma / \pi \varepsilon_0 f \qquad (6)$$

where f is the applied frequency, ε_0 is the relative complex permittivity of vacuum. From the equation (6), it is clearly that the ε'' is proportional to σ. Based on the free electron theory, the decreased ε'' value is caused by the reduced σ value. Meanwhile, when the ε'' declines, the corresponding ε' value also presents the decreased tendency although the detailed association is still unclear. On the basis of above analysis, the varied permittivity values as elevated temperatures can be easily understood.

Supplementary Note 3. Analysis of graphitized degree of carbon matrix

As shown in Raman spectra (Supplementary Figs. 15 and 16), the I_D/I_G ratios are estimated to be 0.98, 0.67 and 0.55 for the 1st, 2nd and 3rd Sn@C samples, indicating the improved graphitized degree. This result is consistent with the results of X-ray photoelectron energy spectra (XPS), meaning that 3rd Sn@C reaches the maximum surface areas of graphitized C-C bonds. According to the simulation, a higher graphitized degree of carbon matrix would greatly benefit to the phonon coupling between Sn and C. As a result, it leads to an ultralow lattice thermal conductivity.

Supplementary Note 4. The MD simulation

In the MD simulation, two atomic models were constructed. The first one is composed of a Sn nanoparticle with the α phase embedded in a graphite matrix. The dimensions of the graphite matrix are around 110×60×62 Å and the Sn nanoparticle is approximate in a cubic shape with a side length of 15 Å. To construct this model, a hole with the same shape and size of the Sn nanoparticle is created at the center of graphite by removing C atoms. Afterwards, the Sn nanoparticle is placed in the hole. An interface of 2 Å thickness is then created between the Sn nanoparticle and the surrounding graphite by removing any C atoms that have distances smaller than 2 Å to any Sn atoms. Finally, the composite system is relaxed in the NVT ensemble (constant volume and temperature) at 300 K for 0.1 ns with a time step of 0.5 fs. The Tersoff potential is adopted to describe the covalent bonding interaction of C atoms and the MEAM potential developed by Ko *et al.*[11] is adopted for Sn atoms. The interactions between the C atoms of crystal graphite from different layers and that between C and Sn atoms are modeled as van der Waals interactions and described by the 12-6 Lennard-Jones (LJ) potential. The corresponding LJ parameters for the C-C and C-Sn atom pairs are adopted from previous works[12,13], respectively. Supplementary Fig. 20a. shows the atomic configuration of this model after the atomic relaxation. The second model is created based on the first one by transferring the graphite from the crystal phase to the amorphous phase using the quenching method. Specifically, the crystal graphite containing the Sn nanoparticle is placed in the NVT ensemble with periodic boundary conditions applied along the three directions. The Sn nanoparticle is fixed as rigid and not allowed to move while the C atoms remain flexible. The system is first rapidly increased to 8000 K in a period of 0.1 ns. Then, the system is held at this temperature for another 0.1 ns such that the crystal graphite is fully melt. Afterwards, the system is cooled step by step with a

temperature decrease of 1000 K at the cooling speed of 20 K/ps. At the end of each cooling step, an atomic relaxation is performed for 0.1 ns at the target temperature of this step. To calculate thermal conductivity of the composite structure of the graphite and Sn nanoparticle, the non-equilibrium method based on the Fourier's law of thermal transport is adopted. The system is first equilibrated at 300 K in the NVT ensemble for 0.1 ns. Then the system is switched to the NVE ensemble (constant volume and energy) and the atoms in the regions on the left and right ends (shed in red) with a thickness of 2 Å are fixed. The atoms in the region shed in blue on the left with a thickness of 5 Å is kept at 300 K, while the atoms in the blue region with the same thickness on the right is kept at a higher temperature of 350 K. Afterwards, the simulation is conducted for 0.1 ns to establish a stable temperature profile along the system length direction. Upon the realization of the stable non-equilibrium state, the simulation is conducted for 0.5 ns to obtain the time-averaged temperature profile. According the Fourier's law, the thermal conductivity can be determined through the formula $J = -\lambda \nabla T$, with J denoting the heat flux and ∇T is the temperature gradient along the heat flux direction. On the other hand, the heat flux J can be obtained by $J = \Delta\xi/(2A\Delta t)$. Here, $L = 1.5 + ex\left(-\frac{|S|}{116}\right)$ is the energy added to the hot bath and subtracted from the two cold baths at each time step and A is the cross-section area.

For further analysis, the phonon spectra *P(ω)* of the Sn nanoparticle and the C atoms near the graphite-Sn interface are calculated. The phonon spectrum *P(ω)* is calculated by performing the fast Fourier transform on the velocity auto-correlation function as:

$$P(\omega) = \frac{1}{\sqrt{2\pi}} \int_0^{\infty} \mathrm{e}^{\mathrm{i}\omega t} \langle \sum_{j=1}^{N} v_j(t) v_j(0) \rangle \, \mathrm{d}\omega \qquad (7)$$

where $v_j(t)$ denotes the velocity of the atom j at time t. The ensemble average in the above equation is realized by time averaging over a period of 50 ps with the sample velocities extracted from the simulation every 5 fs. The calculation results show that the inserted numbers, sizes and graphitized degree have a greatly in the thermal conductivity. The calculation data are presented in Supplementary Fig. 15. Based on these results, the conclusions can be made as follows:

(1) The implant of Sn nanoparticle in amorphous carbon almost does not influence its thermal conductivity, even if changing the size (1~3 nm) and numbers (1~3 in one unit). However, for the crystal graphite, its thermal conductivity is significantly reduced after the inserted by Sn nanoparticle. This dramatic reduction is mainly caused by the phonon scattering between graphitized carbon atom and Sn NPs.

(2) In one graphitized carbon unit, a larger size or increased the number of inserted Sn NPs would reduce the thermal conductivity sharply.

Supplementary Note 5. Calculations of the κ_e and κ_l:

To better understand the mechanism of thermal conductivity (κ_T) for the device, the lattice and electron thermal conductivity can be calculated based on the Widemann-Franz relationship[14]:

$$\kappa_T = \kappa_e + \kappa_L \tag{8}$$

$$\kappa_e = \sigma \times L \times T \tag{9}$$

where L presents the Lorenz number. Commonly, L has a correlation with the S value, as described follows:

$$L = 1.5 + ex\left(-\frac{|S|}{116}\right) \tag{10}$$

where L is in units of 10^{-8} W ΩK^{-2} and α in μV/K. Based on above equations (8−10), the κ_e and κ_L can be obtained at rising temperature.

Supplementary Note 6. The influence of PL thickness on the EM-harvesting ability

In Supplementary Fig. 18, we observed that the PL layer thickness increased as increasing the amount of parylene monomers. When 0.25, 0.5, 0.75 and 1.0 g of parylene amounts were provided, the thicknesses of PL layers were estimated to be 210, 390, 460, and 680 nm, respectively. The varied PL layer greatly influences the RMS, as shown in Supplementary Fig. 17a. Specifically, the RMS decreases sharply as increasing the PL layer and then keeps a constant value after reaching the maximum value (corresponding thickness ~460 nm). Subsequently, the frequency dependent of EM dissipation factor (η) of the Sn@C/PL film was studied in Supplementary Fig. 17b. It could be found that the η showed the opposite trend, as comapred to the RMS. When thickness equaled to 460 nm, we observed that η values would become maximum value. But further increases to 680 nm, the cofficents presents a tiny decrease. An increased coefficient η may be attributed to the reduced RMS and existed RL layer. Specifically, the PL layer showed an excellent wave-transparent ability, as compared to the Sn@C film without PL layer, which was favorable to a smaller electromagnetic reflection. Additionally, further reduction occurs on a surface with smaller RMS value, according to wave-scattering thoery.

Supplementary Note 7. EM simulation

To demonstrate higher EM harvesting performance of the proposed multilayer structure (Au pattern/Sn@C/PL), the full wave simulation was performed using a commercial software CST Microwave Studio 2016. Note that the top layer is the dielectric substrate with the thickness of 460 nm, the middle layer serves as the absorption layer (total thickness 5.0 μm, and the third layer is the periodic Au patches with the thickness of ~50 nm. To carry out the simulated transmission performance,

the typical structure is built in CST Microwave studio and placed in the unit cell boundary condition, which simulate the case where the size of the proposed structure is infinite. In the meantime, Floquet port with the E-field along the long side of the Au particles (W_1=0.065 cm; W_2=0.36 cm) is set as the excitation source. Therefore, from the simulated S parameters (S_{21}), it is easy to find that nearly no transmission energy can be observed from 2.0 to 8.0 GHz when illuminated with the normal incidence, thereby demonstrating the excellent EM harvesting ability of the proposed design.

Supplementary Note 8. Heat-resistance of Sn@C/PL leg

Usually, the as-designed EM-electricity harvester needs to be used in medium temperature region. Hence, an excellent heat-resistance is requested here to ensure the stability. Supplementary Fig. 22 displayed the time dependent of surface conditions of the device after heated at 523 K (a little greater than the maximum proposed temperature, 473 K). The measured RMS value of device slowly increased from 49 to 64 nm as increasing the time to 12 h. The slightly increased RMS values might be attributed to the different thermal expansion coefficients between Sn@C interlayer and PL layer. Even so, these key coefficients, especially permittivity parameters only possessed a tiny fluctuation after heated for 12 h.

Supplementary Note 9. Analysis of thermal-gradient under applied EM radiation

To illustrate the connection between temperature gradient and wave harvesting, the homemade microwave cavity was employed to radiate the Sn@C leg. The temperature difference between the PL covered area and other area was recorded at different microwave radiation time. Note that the operation frequency of microwave oven (f = 2.45 GHz) ranges in our proposed frequency region. Concerning the EM

safety, the ΔT value was recorded after completing the microwave radiation. As shown in Supplementary Fig. 24, it can be clearly observed that the temperature-gradient between PL covered area and other area gradually increases after rising the radiation time from 0 to 150 s. After extending the time to 180 s, the ΔT exhibits a tiny decrease. The generation of temperature difference can be explained as following:

i) The PL covered area has a higher η value, thus more incident EM energy would be absorbed and then yield heats.

ii) The front of partial Sn@C film is covered by the PL layer, showing a ultralow thermal conductivity (~0.07 Wm/K). Therefore, it could avoid the quick disspiation of heats and a distinct temperature-rising can be observed. While for the area with uncovered PL layer, the generated heats would quick diffuse to the sourroundings. In this case, a desirable ΔT would be formed between PL-covered and uncovered area.

Supplementary Note 10. Flexibility analysis

Before the flexibility testing, the device was firmly attached onto the surface of a rod-like glass tube. The changes of bending angle could be realized by tuning the attached area. In our case, the PL monomer with high surface energy can be easily absorbed on the surface of Sn@C layer, which was benefited to the release of strain. As a result, the device exhibited an excellent flexibility.

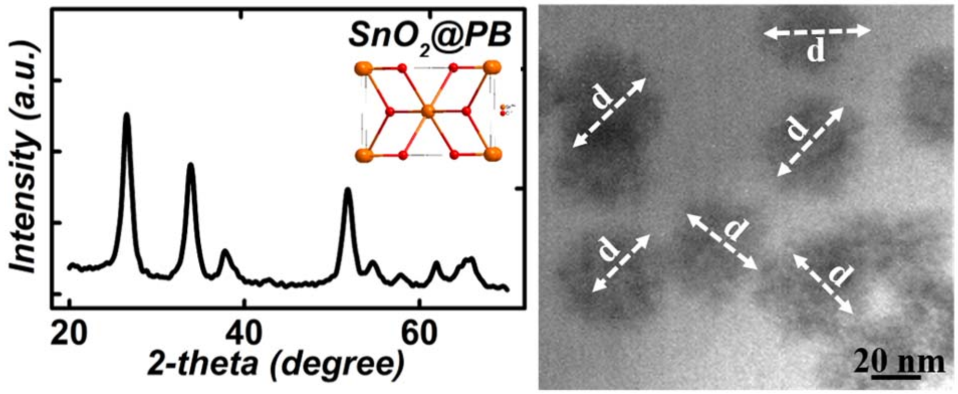

Supplementary Figure 1. XRD pattern and low-magnification TEM image of SnO_2@PB sample.

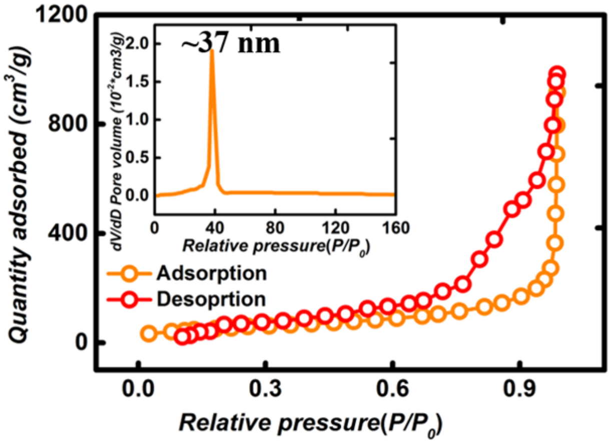

Supplementary Figure 2. N_2 adsorption-desorption isotherm for the Sn@C composites prepared with 3rd cycled annealing treatment. Inserted image showing the pore size distribution.

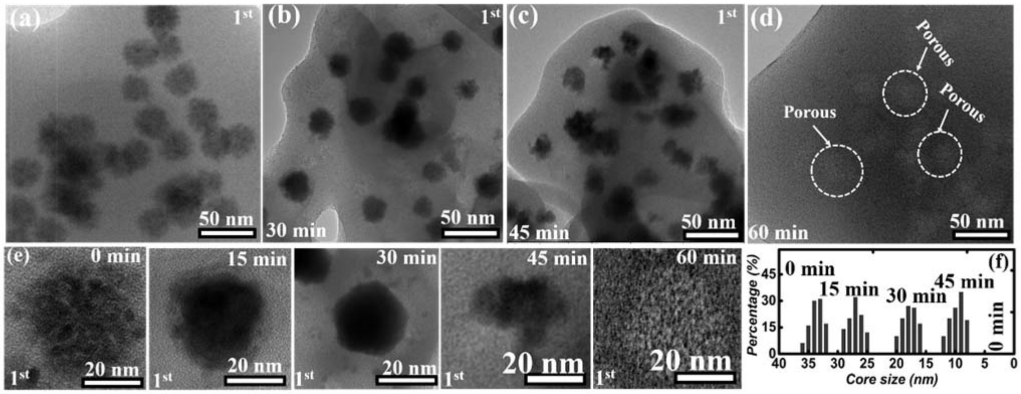

Supplementary Figure 3. (a-d) TEM images of the SnO_2@PB sample treated at various times. (e) TEM images of SnO_2 core-evolution gained at various times. (f) The average size distribution of SnO_2 core after treated at various times.

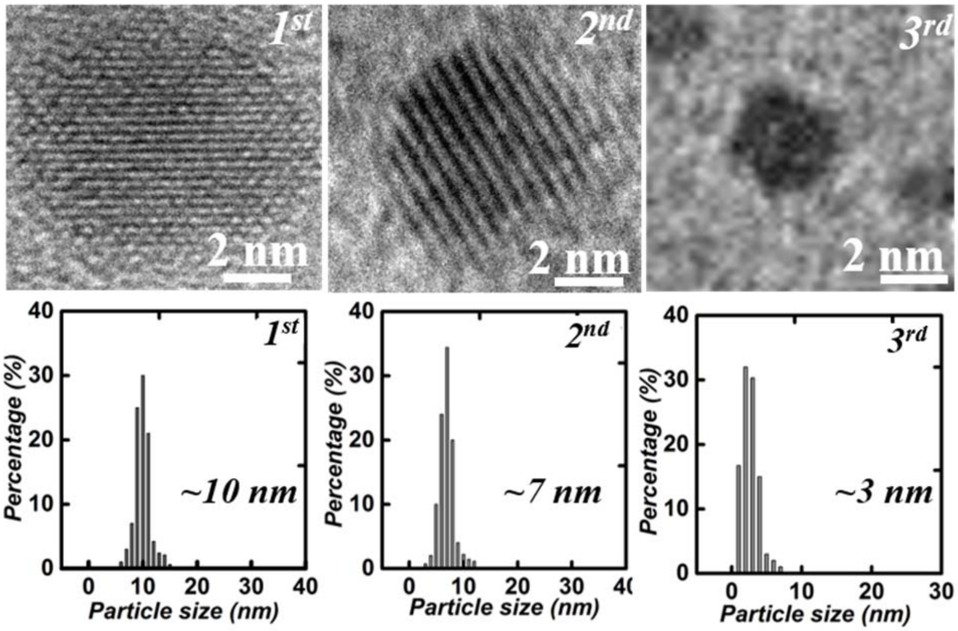

Supplementary Figure 4. The varied sizes of Sn NPs gained with cycled annealing treatment.

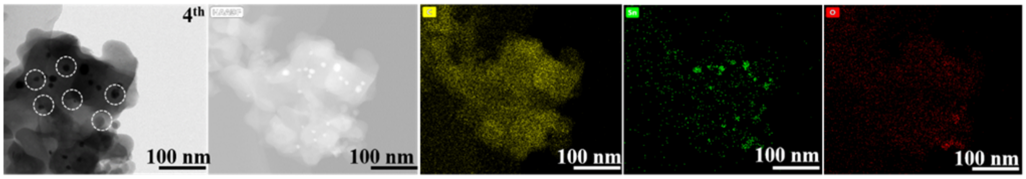

Supplementary Figure 5. HADDF-STEM images of the Sn@C sample obtained with 4th cycled annealed treatment.

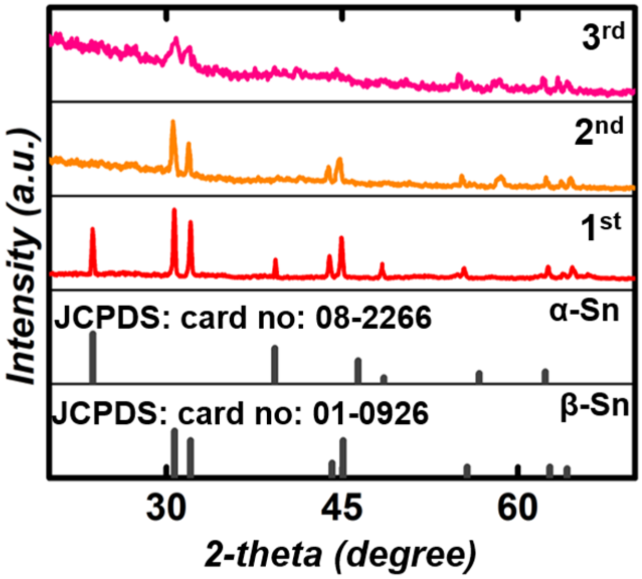

Supplementary Figure 6. XRD patterns of the Sn@C samples obtained with cycled annealing treatment.

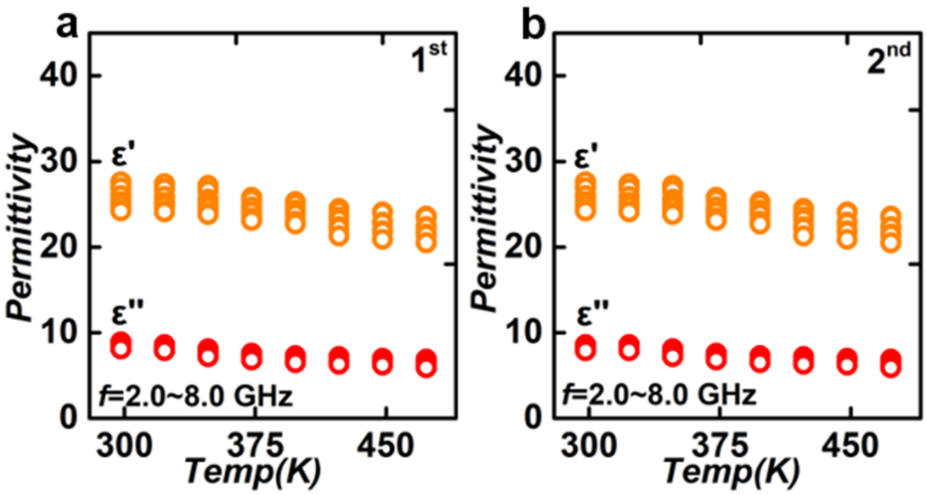

Supplementary Figure 7. Temperature dependency of permittivity for the 1st and 2nd Sn@C measured at 2~8.0 GHz.

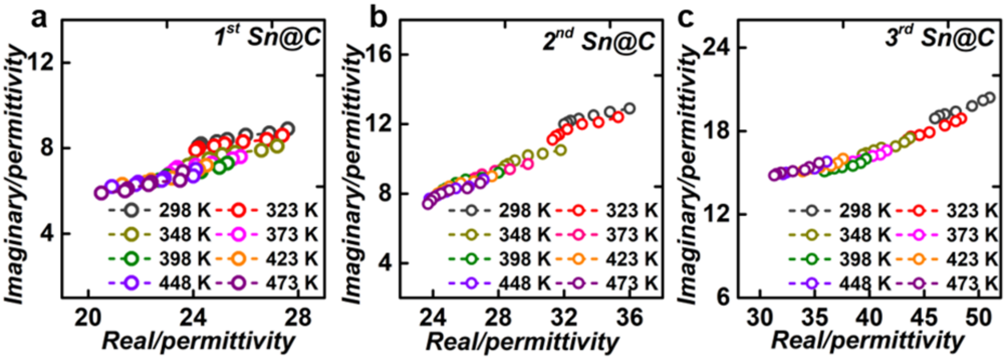

Supplementary Figure 8. Cole-Cole curves for these Sn@C samples made with cycled annealing treatment: (a) 1st Sn@C; (b) 2nd Sn@C; (c) 3rd Sn@C (frquency ranging from 2 to 8.0 GHz)

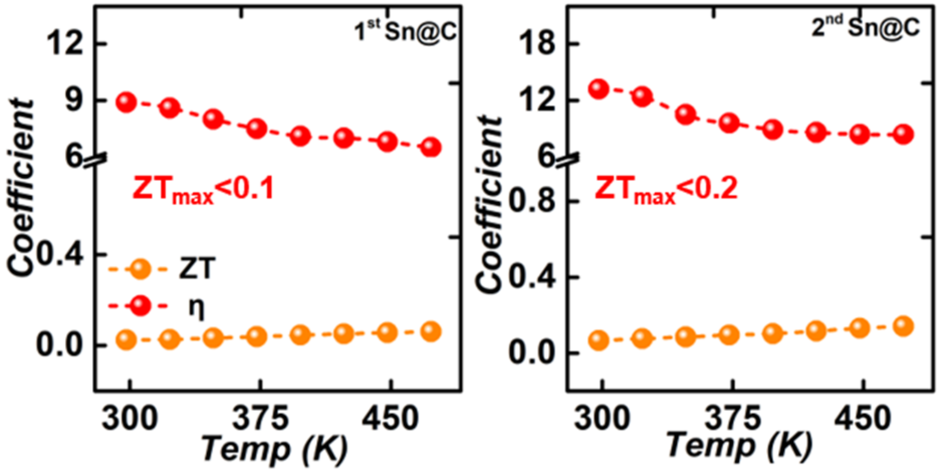

Supplementary Figure 9. Anlysis of coupled EM-electricity performance for 1st and 2nd Sn@C (note the the η value exhibits a tiny fluaction at 2~8.0 GHz, hence the average η vaue used here to stand for the dielectric dipisspation ablity at whole 2-8.0 GHz).

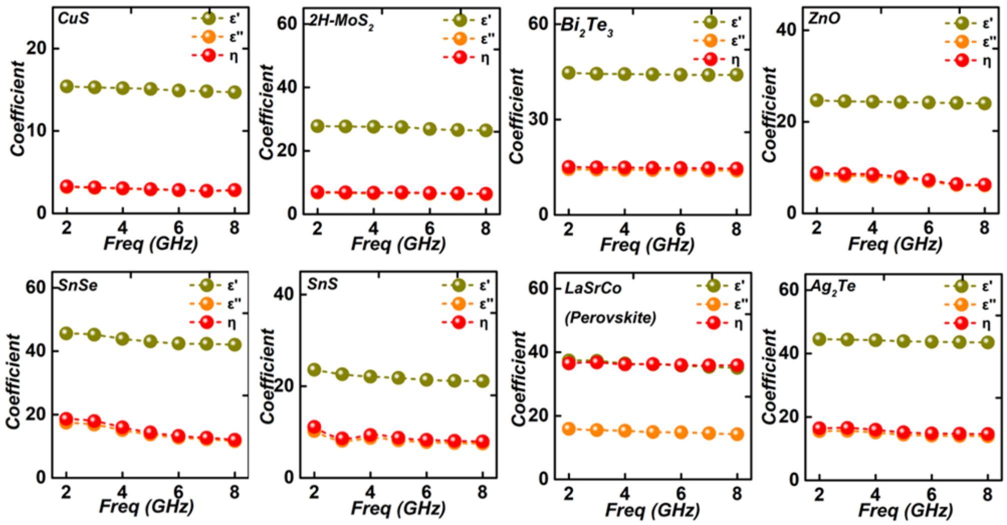

Supplementary Figure 10. The permittivity and EM disspipaton factor of commonly investigated TE materials.

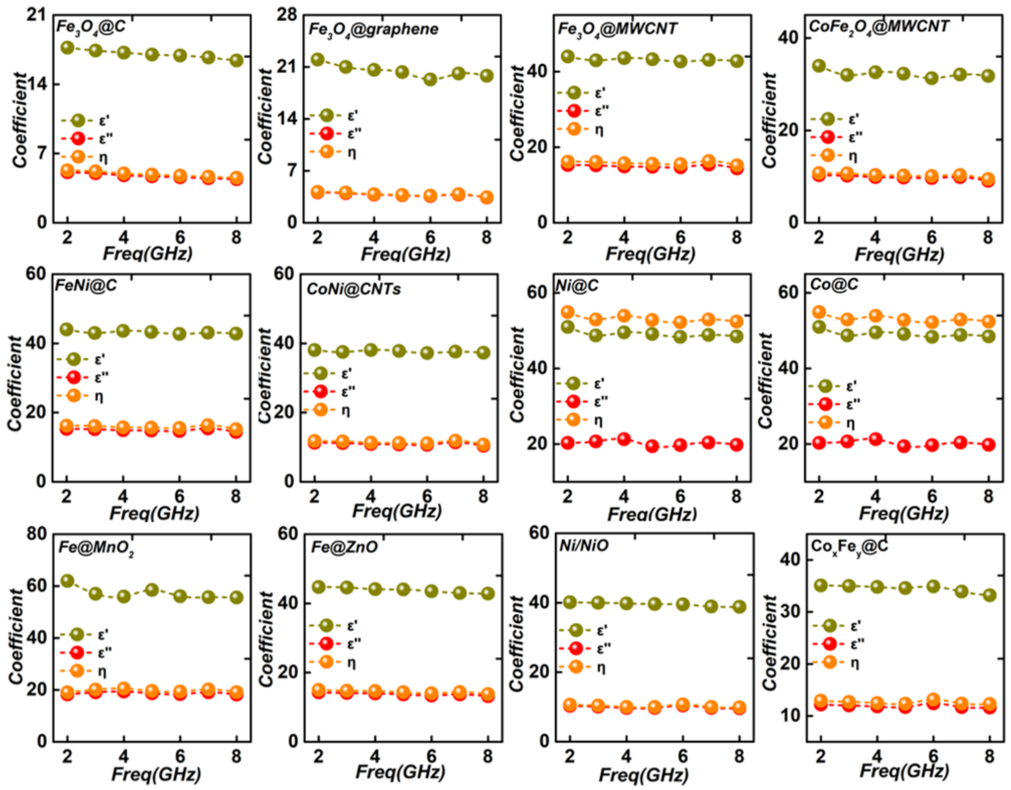

Supplementary Figure 11. The permittivity and EM disspipaton factor of commonly investigated EM shielding or absorbing materials.

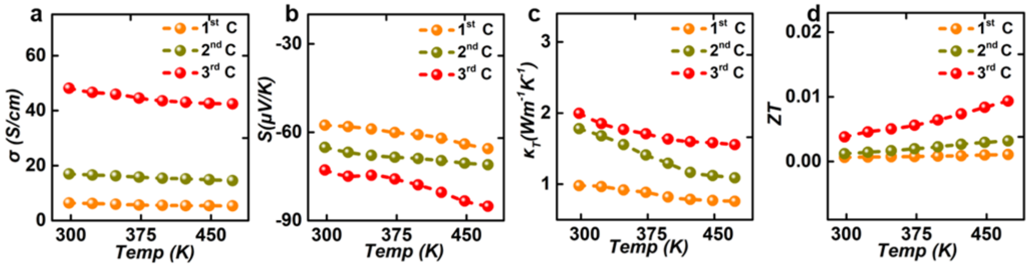

Supplementary Figure 12. The temperature dependent of conductivity (σ), thermal conductivity (κ_T), seebeck coefficient (*S*) and *ZT* value (Sn NPs have been removed by HCl solution). Note that the pure carbon poessess the poor *ZT* values, thus cannot be used for the purpose of EM-electricity coversion.

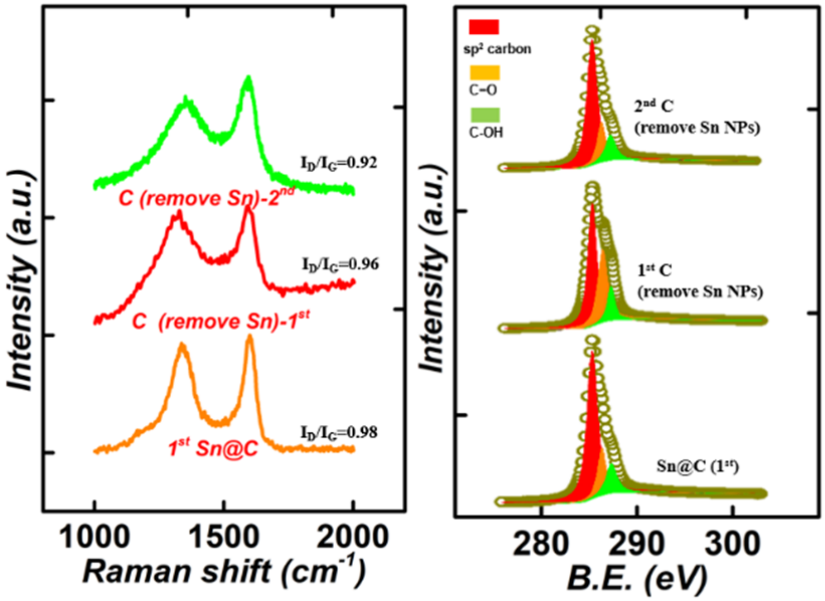

Supplementary Figure 13. Raman spectra (left) and X-ray electron energy spectra (right) of Sn@C sample (1st annealed treatment) and porous carbon matrix only ($1\text{-}2^{nd}$ annealed treatment).

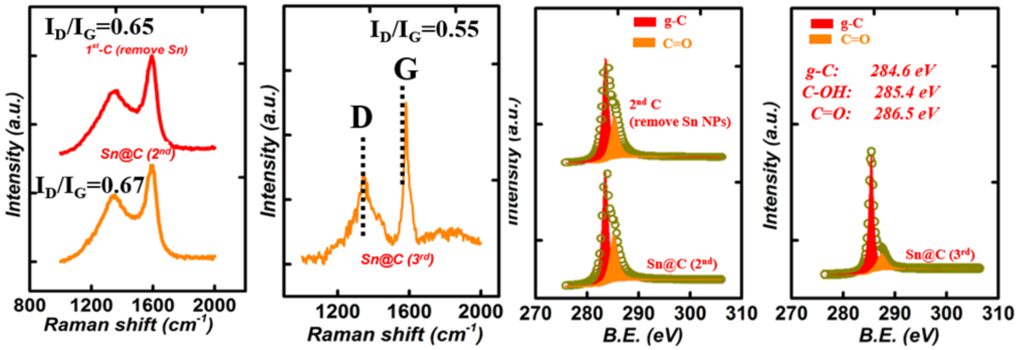

Supplementary Figure14. Raman spectra and X-ray electron energy spectrum (XPS) of Sn@C sample (under 2nd and 3rd annealing treatment) and corresponding porous carbon matrix (1st and 2nd annealing treatment). After removing the Sn NPs, it can be found that the pure carbon matrix shows no significant variation on graphtization level, even if conducting a cycled heating treatment.

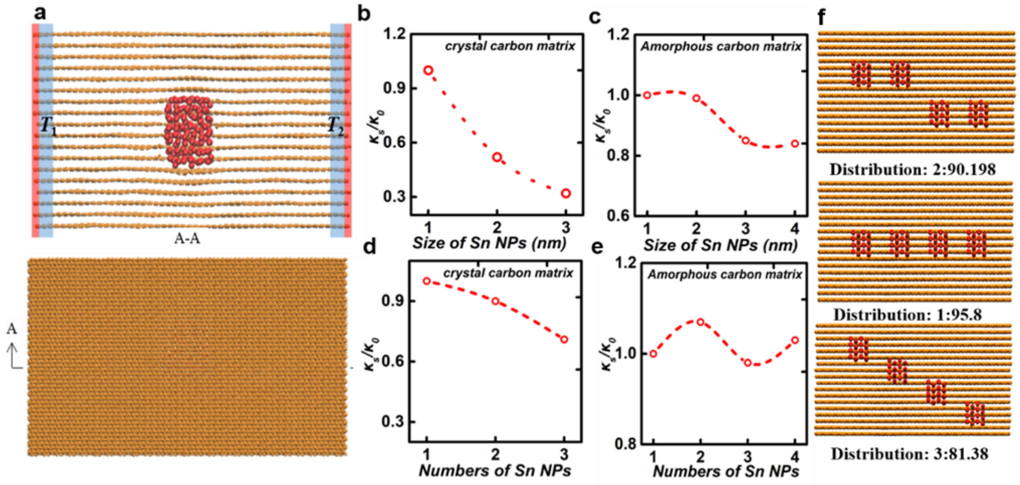

Supplementary Figure 15. MD simulation: (a) illustration of the atomic configuration of the model after the atomic relaxation. (b-e) The ratio (κ_s/κ_0) as a function of numbers, sizes of Sn nanoparticles in crystal or amorphous carbon matrix; (f) the detailed distribution state of the inserted Sn NPs.

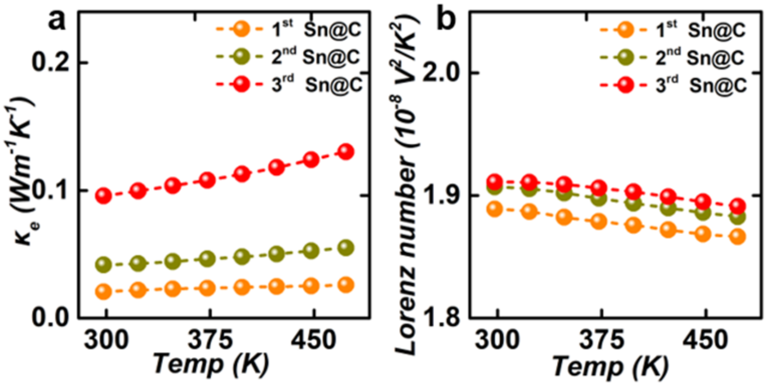

Supplementary Figure 16. (a) Temperature-dependent Lorenz number (L) based on a multiband model and (b) temperature-dependent electron thermal conductivity (κ_e).

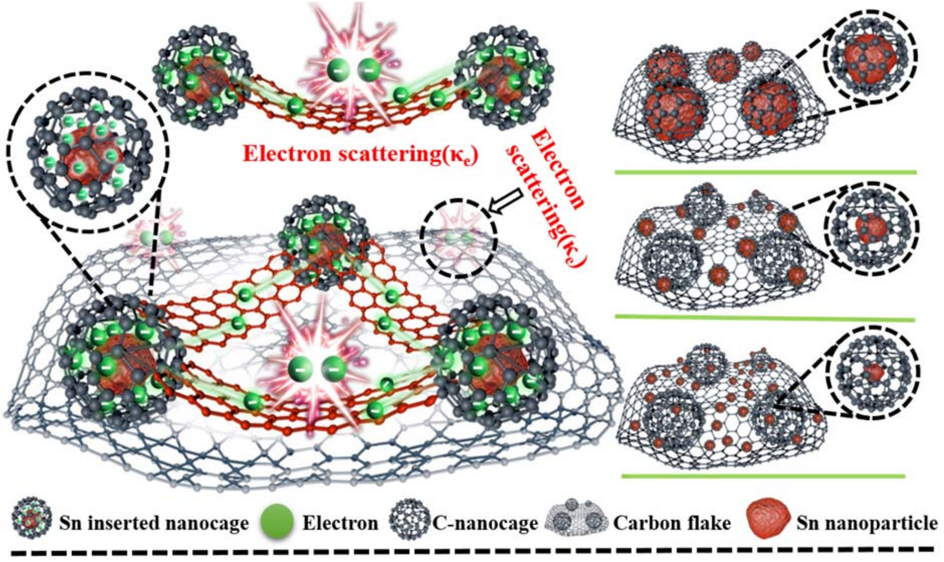

Supplementary Figure 17. Schematic illustration to explain the conflict between σ and κ_e: the conductivity sharply increases, attributing to the phase conversion from α to β-Sn, reduced grain boundaries and highly graphitization of carbon matrix after Sn splitting. The increased σ did not change the κ_e significantly, mainly be ascribed to the unique structure with Sn inserted carbon flake, which provides more channels for the electrons transport. Consequently, the electron scattering is weak, even if the carrier concentration is improved.

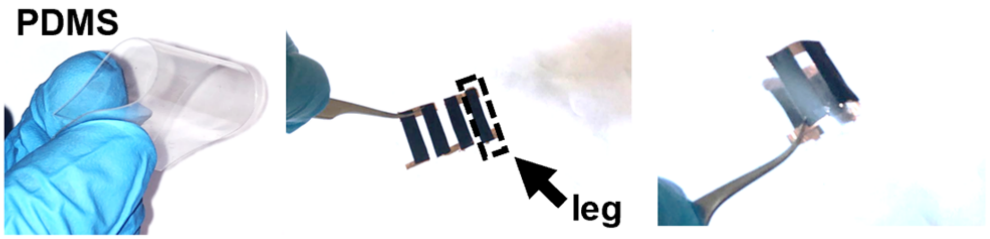

Supplementary Figure 18. Photograph of the PDMS substrate and the as-fabricated EM-electricity device.

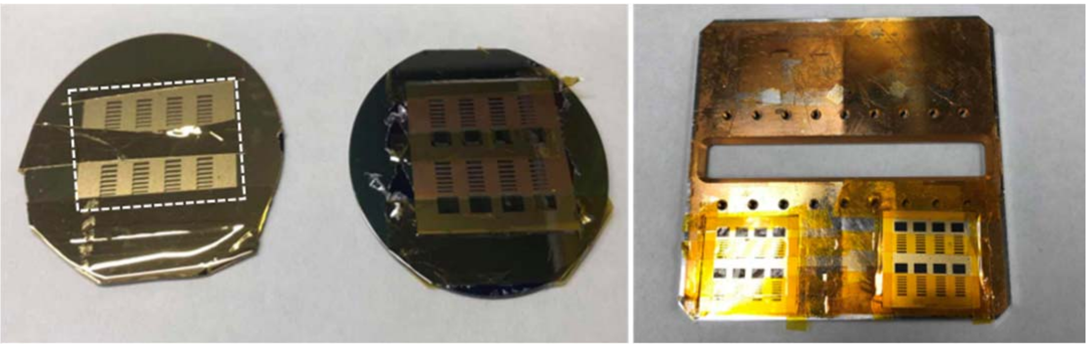

Supplementary Figure 19. Digital images of mask for the preparation of Au pattern.

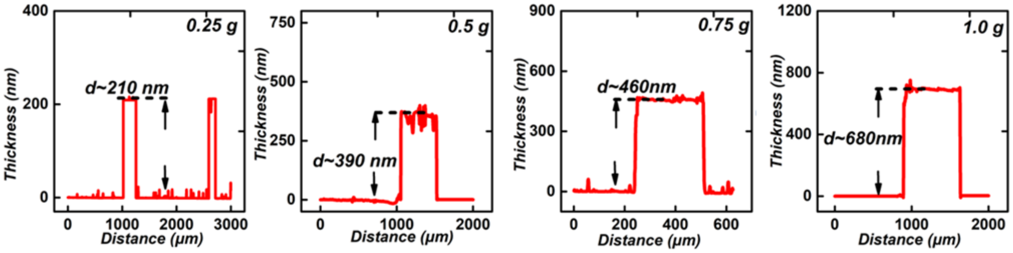

Supplementary Figure 20. The deposited PL thickness prepared with various of Dix-c amount (parylene as the monomer).

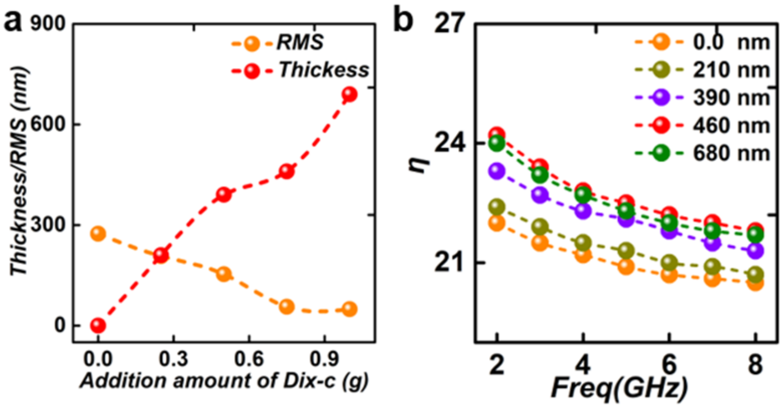

Supplementary Figure 21. The influence of PL thickness on dielectric dissipation: (a) the RMS and thickness of PL layer with various of Dix-c; (b) the absorption coefficient for the film made with the top and interlayer. (Here the film does not contain the bottom layer, in case to inverstigate the effect of PL layer on absorption coefficient).

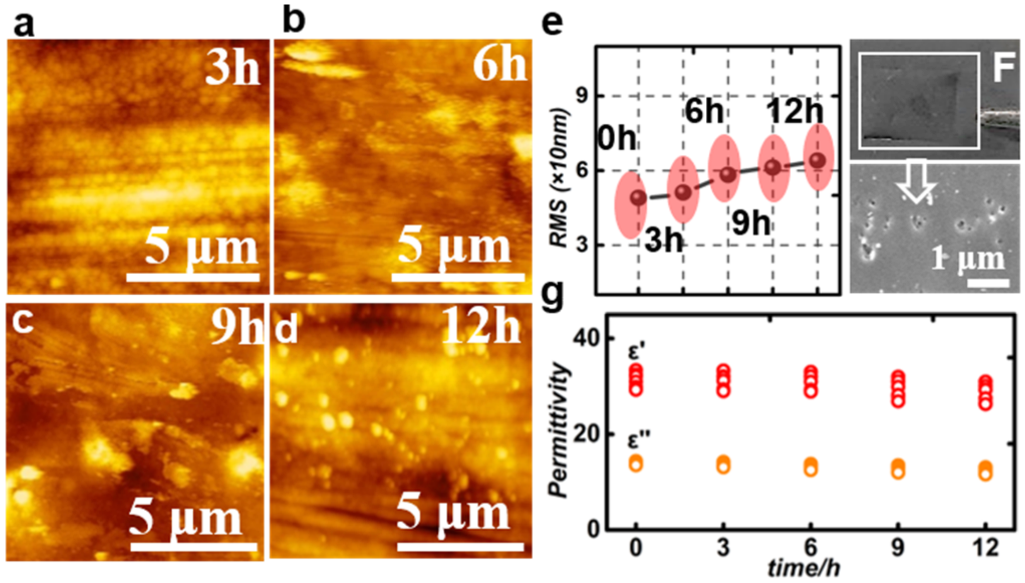

Supplementary Figure 22. Heat-resistance performance: (a-d) AFM images of the Sn@C/PL leg heated at 523 K and then tested at every 3h; (e) RMS values of Sn@C/PL leg heated with various hours (heated at 523 K); (f) FESEM images of the Sn@C/PL leg before and after heating with 12 hours; (g) time-dependent of permittivity values for Sn@C/PL leg.

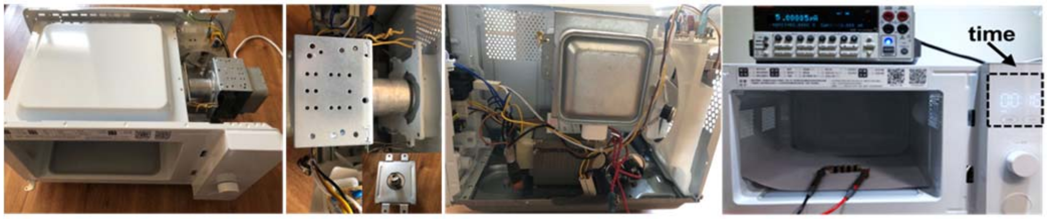

Supplementary Figure 23. Homemade microwave cavity and apparatus for the performance measurement. Note that the magnetron of microwave oven has a large power (exceeding 1000 W) and sealed condition is requested to conduct microwave oven. In our case, we have changed the electronic circuit which the EM captivity can be used in an open stage. Meanwhile, the original magnetron (~1200 W) was replaced to a new one with lower power (~100 W).

.

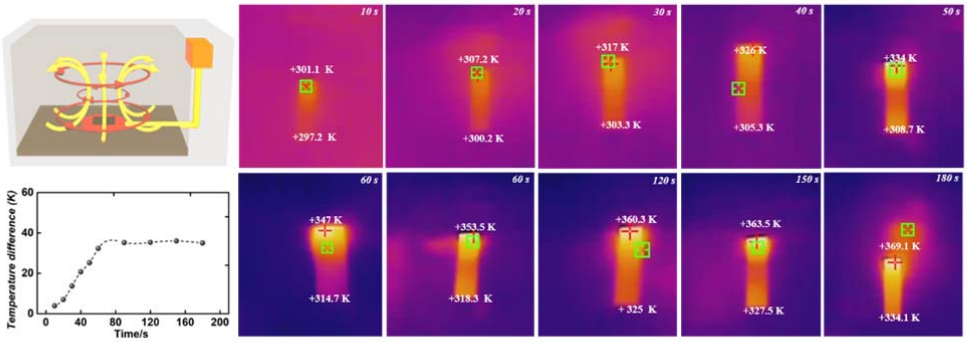

Supplementary Figure 24. Time dependent of temperature difference for the EM-electricity device after microwave radiating for 0~180 s.

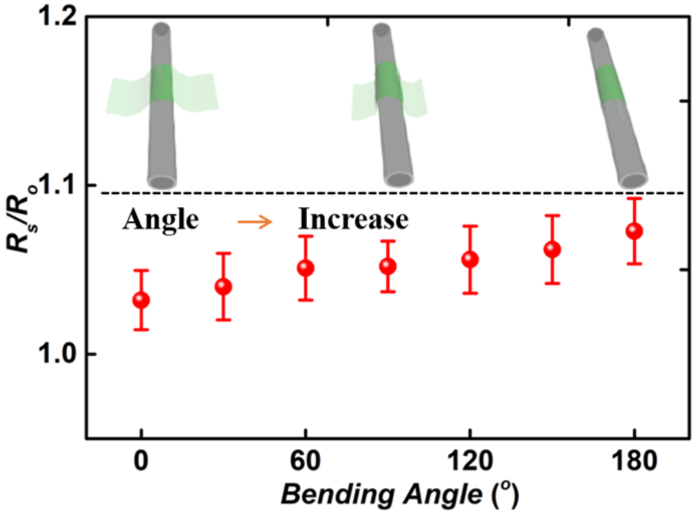

Supplementary Figure 25. The resistance (R_s) as a function of bending angle (θ) for the optimal EM-electricity device. Each point shows the standard deviations from three independent measurements

Supplementary Table 1. Comparison of the *ZT* values of current work with the recently reported liteatures.

Samples	ZT_{max}	T_{max} (K)	Flexible or not	ZT at 473 K	Refs.
PbSe/CdSe	1.4	873	Not	<0.4	15
$Ta_{1-x}Ti_xFeSb$	1.52	973	Not	<0.4	16
$SnS_{0.91}Se_{0.09}$	1.6	873	Not	<0.5	17
$PbSe_{0.85}Te_{0.15}$	1.7	900	Not	<0.5	18
$Cu_{12}Sb_4S_{13}$	1.2	723	Not	<0.4	19
$PbSe/NaSbSe_2$	1.4	900	Not	<0.4	20
$AgBi_3S_5$	1.0	800	Not	<0.3	21
Bi dotted PbTe	1.35	675	Not	<0.5	22
$Cu_{2-y}Se_{0.5}S_{0.5}$	2.3	1000	Not	<0.4	23
Bi/Bi_2S_3	0.36	623	Not	<0.2	24
$ZrNiPb_{0.98-x}Sn_xBi_{0.002}$	~0.72	900	Not	<0.4	25
$Sn_{1-x}Na_x$	0.65	850	Not	<0.1	26
3rd Sn@C	0.62	473	Yes	0.62	This work

Supplementary Table 2. Comparison of our EM-electricity harvester with the recently reported devices.

Samples	Output voltage (mV)	Output power (nW/cm^2)	Temperature difference (K)	Refs.
Te/poly(3-hexylthiophene)	38.0	N.A.	55	27
Bi_2Te_3	520	N.A.	50	28
MoS_2/PU	~1.8	N.A.	N.A.	29
$Ag_2Se/Ag/CuAgSe$	12.2	488	45	30
PEI-MWCNTs	58	650	50	31
TiS_2/organic	N.A.	2.5	70	32
EM-electricity harvester	14.3	3.94	36	This work

Supplementary Table 3. The detailed parameters for the cycled annealing treamtment.

Samples	heating rate	Annealing time	Atmosphere	Annealing temperature	Cooling method	Size of Sn NPs
1st Sn@C	5 ^{o}C/min	2.0 h	H_2/N_2	900 ^{o}C	Natural cooling	~10 nm
2nd Sn@C	10 ^{o}C/min	1.0 h	N_2	750 ^{o}C	water	~7 nm
3rd Sn@C	15 ^{o}C/min	0.3 h	N_2	750 ^{o}C	water	~3 nm
4th Sn@C	20 ^{o}C/min	0.3 h	N_2	750 ^{o}C	water	2~20 nm

Supplementary References

[1] Chen, J.B.; Liang, X.G.; Quan, B.; Yang, Z.H.; Du, Y.W.; Ji, G.B. 3D Flake-like Bi_2Te_3 with outstanding lightweight electromagnetic wave absorption feature. *Part. Part. Sys. Charact.* **35**, 1700468 (2018).

[2] Zuo, P.; Zhang, S.Y.; Jin, B.K.; Tian, Y.P.; Yang, J.X. Rapid synthesis and electrochemical property of Ag_2Te nanorods. *J. Phys. Chem. C* **112**, 14825−14829 (2008).

[3] Lv, H.; Ji G.B.; Liang, X.G.; Zhang, H.Q.; Du, Y.W. A novel rod-like MnO_2@Fe loading on graphene giving excellent electromagnteic absorption properties. *J. Mater. Chem. C* **3**, 5056−5064 (2015).

[4] Zhang, Y.Q.; Guo, B.S.; Hu, L.Y.; Xu, Q.J.; Li, Y.; Liu, D.Y.; Xu, M.W. Synthesis of SnS nanoparticle-modifed Mxene($Ti_3C_2T_x$) composites for enhanced sodium storage. *J. Alloys Compd.* **732**, 448−453 (2018).

[5] Yang, X.; Zhang, R.Y.; Chen, N.; Meng, X.; Tang, P.L.; Wang, C.Z.; Zhang, Y.Q.; Wei, Y.J.; Du. F. Assembly of SnSe nanoparticles confined in graphene for enhanced sodium-ion storage performance. *Chem. Eur. J.* **22**, 1445−1451 (2016).

[6] Guo, Y.H.; Dong, S.T.; Liu, S.W.; Cheng, Y.; Zhang, Z.Y.; Wang, H.Y. Fabrication of porous disk-like Ni/NiO microwave absorber and tis excellent broad frequency absorption performance. *J. Alloys Compd.* **731**, 143−149 (2018).

[7] Lv, H.L.; Guo, Y.H.; Yang, Z.H.; Cheng, Y.; Wang, P.L.Y.; Zhang, B.S.; Zhao, Y.; Xu, J.Z.C.; Ji, G.B. A brief introduction to the fabrication and synthesis of graphene based composites for the realization of electromagnetic absorbing materials. *J. Mater. Chem. C* **5**, 491−512 (2017).

[8] Zhang, D.Q.; Xiong, Y.F.; Cheng, J.Y.; Chai, J.X.; Liu, T.T.; Ba, X.W.; Ullah, S.; Zheng, G.P.; Yan, M.; Cao, M.S. Synergetic dielectric loss and magnetic loss towards superior micrwoave absorption through hybridization of few-layer WS_2 nanosheets with NiO nanoparticles. *Sci. Bull.* **65**, 138−146 (2020).

[9] Liu, P.B.; Zhu, C. Y.; Gao, S.; Guan, C.; Guan, C.; Huang, Y.; He, W.J.. N-doped porous carbon nanoplates embedded with CoS_2 vertically anchored on carbon cloths for flexible and ultrahigh microwave absorption. *Carbon* **163**, 348−359 (2020).

[10] Wu, G.L.; Jia, Z.R.; Zhou, X.F.; Nie, G.Z.; Lv, H.L.; Interlayer controllable of hierarchical MWCNTs@C@Fe_xO_y cross-linked composite with wideband electromagnetic absorption performance. *Compos. Part A-Appl. S.* **128**, 105687(2020).

[10] Tersoff, J. Modeling solid-state chemistry-interatomic potentials for multicomponent systems. *Phys. Rev. B*. **39**, 5566−5568 (1989).

[11] Ko, W.S.; Kim, D.H.; Kwon, Y.J.; Lee, M.H. Atomistic simulations of pure tine based on a new modified embedded-atom method interatomic potentials.

Metals-Basel: **8**, 900 (2018).

[12] Girifalco, L.A.; Hodak, M.; Lee, R.S. Carbon nanotube, buckyballs, ropes and a universal graphitic potential. *Phys. Rev*. B. **62**, 13104 (2000).

[13] Hong, Y.; Zhu, C.Q.; Ju, M.G.; Zhang, J.C.; Zeng, X.C. Lateral and flexural phonon thermal transport in graphene and stanine bilayers. *Phys. Chem. Chem. Phys.* **19**, 6554−656 (2017)

[14] Shi, X.L.; Zhou, J.; Chen, Z.G. Advanced thermoelectric design: from materials and structures and structures to devices. *Chem. Rev*. **120**, 7399−7515 (2020).

[15] Qian, X.; Wu, H.J.; Yang, D.Y.; Zhang, Y.; Wang, J.F.; Wang, G.T.; Zhang, L.; Pennycook, S.J.; Zhao, L.D. Synergistically optimizing interdependent thermoelectric parameters of *n*-type PbSe through alloying CdSe. *Energy Environ. Sci.* **12**, 1969−1978 (2019).

[16] Zhu, H.T.; Mao, J.; Li, Y.W.; Sun, J.F.; Wang, Y.M.; Zhu, Q.; Li, G.B.; Song, Q.C; Zou, J.W.; Fu, Y.H.; He, R.; Tong, T.; Liu, Z.H.; Ren, W.Y.; You, L.; Wang, Z.W.; Luo, J. Sotnilkov, A.; Bao, J.M.; Nielsch, K.; Chen, G.; Singh, D.J.; Ren, Z.F.; Discovery of TaFeSb-based half-Heuslers with high thermoelectric performance. *Nat. Commum.* **10**, 270−277 (2019).

[17] He, W.; Wang, D.Y.; Wu, H.J.; Xiao, Y.; Zhang, Y.; He, D.S.; Feng, Y.; Hao, Y.J.; Dong, J.F.; Chetty, R.; Hao, L.J.; Chen, D.F.; Qin, J.F.; Yang, Q.; Li, X.; Song, J.M.; Zhu, Y.C.; Xu, W.; Niu, C.L.; Li, X.; wang, G.T.; Liu, C.; Ohta, M.; Pennycook, S.J.; He, J.Q.; Li, J.F.; Zhao, L.D.High thermoelectric performance in low-cost $SnS_{0.91}Se_{0.09}$ crystals. *Sciences* **27**, 1418−1424 (2019).

[18] Tan, G.J.; Hao, S.Q.; Cai, S.T.; Bailey, T.P.; Luo, Z.Z.; Hadar, I.; Uher, C.; David, V.P.; Wolverton, C.; Kanatzidis, M.G. All-scale hierarchically structured *p*-type PbSe alloys with high thermoelectric performance enabled by improved band degeneracy, *J.*

Am. Chem. Soc. **141**, 4480−4486 (2019).

[19] Sun, F.H.; Dong, J.F.; Tang, H.C.; Shang, P.P. Zhuang, H.L.; Hu, H.H.; Wu, C.F.; Pan, Y.; Li, J.F. Enhanced performance of thermoelectric nanocomposites based on $Cu_{12}Sb_4S_{13}$. *Nano Energy* **57**, 835−841(2019).

[20] Slade, T.J.; Bailet, T.P.; Grovogui, J.A.; Hua, X.; Zhang, X.M.; Kuo, J.J.; Hadar, I.; Snyder, G.B.; Wolverton, C.; David, V.P.; Uher.; Kanatzidis M.G. High thermoelectric performance in PbSe-$NaSeSe_2$ alloys from valance band convergence and low thermal conductivity. *Adv. Energy. Mater.* **9**, 1901377 (2019).

[21] Tan, G.J.; Hao, S.Q.; Zhao, J.L Wolverton, C.; Kanatzidis, M.G. High thermoelectric performance in electron-doped $AgBi3S_5$ with ultralow thermal conductivity. *J. Am. Chem. Sco.* **139**, 6467−6473 (2017).

[22] Yang, L.; Chen, Z.G.; Hong, M.; Wang, L.H.; Kong, D.L.; Huang, L.Q.; Han, G.; Zou, Y.C.; Dargusch, M.; Zou, J. *n*-type Bi-doped PbTe nanocubes with enhanced thermoelectric performance. *Nano Energy* **31**, 105−112 (2017).

[23] Zhao, K.P.; Qiu, P.F.; Song, Q.F.; Blichfeld, A.B.; Eikeland, E.; Ren, D.; Iversen, B.B.; Shi, X.; Chen, L.D. Ultrahigh thermoelectric performance in $Cu_{2-y}Se_{0.5}S_{0.5}$ liquid-like materials. *Mater. Today Phys*. **1**, 14−23 (2017).

[24] Ge, Z.H.; Qin, P.; He, D.S.; Chong, X.Y.; Feng, D.; Ji, Y.H.; Feng, J.; He, J.Q. Highly enhanced thermoelectric properties of Bi/Bi_2S_3 nanocomposites. *ACS Appl. Mater. Interfaces.* **9**, 4828−4834 (2017).

[25] Mao, J.; Zhou, J.W.; Zhu, H.T.; Liu, Z.H.; Zhang, H.; He, R.; Chen, G.; Ren, Z.F. Thermoelectric properties of *n*-type ZrNiPb-based half-Heuslers. *Chem. Mater.* **29**, 867−872 (2017).

[26] Zhou, B.Q.; Li, S.; Li, W.; Li, J.; Zhang, Z.Y.; Lin, S.Q.; Chen, Z.W.; Pei, Y.Z. Thermoelectric properties of SnS with Na-doping. *ACS Appl. Mater. Interfaces.* **9**,

34033−34041 (2017).

[27] Yang Y.; Lin, Z.H.; Hou, T.C.; Zhang, F.;Wang, Z.L. Nanowire-composite based flexible thermoelectric nanogenerators and self-powered temperature sensros. *Nano Res.* **5**, 888−895 (2012).

[28] Feng, R.; Tang, F.; Zhang, N.; Wang, X.H.; Flexible, high-powder desnsity, wearble thermoelectric nanogenerator and self-powdered temperature sensor. *ACS Appl. Mater. Interfaces* **11**, 38616−38624 (2019).

[29] He, M.H.; Lin, Y.J.; Chiu, C.M.; Yang, W. F.; Zhang, B.B.; Yun, D. Q.; Xie, Y.N.; Lin, Z.H. A flexible photo-thermoelectric nanogenerator based on MoS_2/PU photothermal layer for infrared light harvesting. *Nano Energy* **49**, 588−595 (2018).

[30] Lu, Y.; Qiu, Y.; Cai, K.F.; Ding, Y.F.; Wang, M. D.; Jiang, C.; Yao, Q.; Yao, Q.; Huang, C.J.; Chen, L.D.; He, J.Q. Ultrahigh powder factor and flexile silver selenide-based composite film for thermoelectri devices. *Energy Envron. Sci.* **13**, 1240−1249 (2020).

[31] Choi, J.; Lee, J.Y.; Lee, S.S.; Park, C.R.; Kim, H. High-performance thermoelectric paper based on double carrier-filtering processess at nanowire heterojunctions. *Adv. Energ. Mater*. **6**, 1502191 (2016).

[32] Tian, R.M.; Wan, C.L.; Wang, Y.F.; Wei, Q.S.; Ishida, T.; Yamamoto, A.; Tsuruta, A.; Shin, Shin, Li, S.; Koumoto, K. A solution-prpcessed TiS_2/organic hybrid superlattice film towards flexible thermoelectric devices. *J. Mater. Chem. A* **5**, 546−570 (2017).
