# Supplementary material for: A flexible electromagnetic wave-electricity harvester
Source: Nat Commun. 2021 Feb 5;12:834. doi: 10.1038/s41467-021-21103-9 (PMC7864982; doi:10.1038/s41467-021-21103-9)
Supplement: Supplementary file 2 — Peer Review File [file 41467_2021_21103_MOESM2_ESM.pdf]

Reviewers' comments:

Reviewer #1 (Remarks to the Author):

Authors fabricated three layered sandwich structures for dual functionality: EM shielding and harvesting EM for power generation. The paper is very poorly written and the literature review is incomplete. One significant concern lies in the energy harvesting. Authors proposed to harvest EM energy via Thermometric materials while thermoelectric power generation depends on the thermal gradient. However, it is not clear how the EM can be used to generate the thermal gradient, which is important for the thermoelectric power generation. Of course, it might be easy to simply show the thermoelectric properties in the lab by intentionally controlling the temperature gradient with lab setup, but the connection between temperature gradient and wave harvesting is not clear. The second concern is the EM wavelength. It is not clear what kind of wavelength was used for this research.

Reviewer #3 (Remarks to the Author):

The authors proposed the new energy harvester using an electromagnetic wave-heat-electricity energy conversion material. One of the interesting points is the high thermoelectric properties of the carbon with the Sn nano-particles (beta phase). The biological cell-like behavior the Sn@C through the cycled annealing is also useful information for the nanotechnology researchers. The non-dimensional figure of merit of the flexible composite, 0.72, is really high and interesting, as well as keeping high electromagnetic absorptance.

The difference between the prepared 3 samples (EA-TE-1, EA-TE-2, EA-TE-3) is just annealing times in my understanding. Then, the most number of the smallest Sn nano-particles is made in EA-TE-3. It might be clear that the thermal conductivity is decreased when the phonons scattering centers are increased in the composite.

The molecular dynamics discussion looks isolated. The authors calculated the phonons density of states as shown in Figs.5(d), (e), and they discuss thermal conductivity decrease shown in Fig.5 (f) using the values of phonon coupling. However, we cannot know the quality of carbon in EA-TE-1, 2, 3. It is difficult to understand the thermal conductivity change from the viewpoints of the phonon coupling values.

Reply to Reviewers' comments about the Manuscript (NCOMMS-20-02530)

Reviewer #1: Authors fabricated three layered sandwich structures for dual functionality: EM shielding and harvesting EM for power generation. The paper is very poorly written, and the literature review is incomplete. One significant concern lies in the energy harvesting. Authors proposed to harvest EM energy via Thermometric materials while thermoelectric power generation depends on the thermal gradient. However, it is not clear how the EM can be used to generate the thermal gradient, which is important for the thermoelectric power generation. Of course, it might be easy to simply show the thermoelectric properties in the lab by intentionally controlling the temperature gradient with lab setup, but the connection between temperature gradient and wave harvesting is not clear. The second concern is the EM wavelength. It is not clear what kind of wavelength was used for this research.

Response: We greatly appreciate Reviewer #1's positive comments.

Q1. The paper is very poorly written, and the literature review is incomplete.

Response: The English writing has been polished by a professional language editing service (the certificate has also been provided). Meanwhile, we have updated the latest literatures and we hope that the current version could be satisfied.

Q2. One significant concern lies in the energy harvesting. Authors proposed to harvest EM energy via Thermometric materials while thermoelectric power generation depends on the thermal gradient. However, it is not clear how the EM can be used to generate the thermal gradient, which is important for the thermoelectric power generation. Of course, it might be easy to simply show the thermoelectric properties in the lab by intentionally controlling the temperature gradient with lab setup, but the connection between temperature gradient and wave harvesting is not clear.

Response: Thanks a lot for Reviewer #1's valuable comments. In our work, the developed device has an excellent EM energy-harvesting ability, i.e. converting the incident EM waves into heat by the dielectric loss (termed as ε''). The ε'' is determined by polarization relaxation (ε_p'') and conductive loss (ε_c'') (*Adv. Mater.*, 2016, 28, 486; *Adv. Funct. Mater.*, 2019, 29, 1807624).

Specifically, at an applied EM filed, the electrical energy enables the carriers to overcome the resistance to directional flow, meaning that the energy loss can be termed as conductive loss. Commonly, ε_c'' is related to the conductivity (σ), based on the free-electron theory (*Adv. Sci.*, 2019, 6, 18001057):

$$\varepsilon_c'' = \sigma / \pi \varepsilon_0 f$$

where σ refers to the conductivity, ε_0 is the permittivity value of air, f is the frequency. Based on above equation, the ε_c'' can be improved by enhancing the conductivity, while the ε_p'' mainly arises from various of the local movement of bound charges and the variation of dipole moment in the alternating EM filed. As a result, both ε_p'' and ε_c'' would dissipate the EM energy, leading to the temperature rise (*Small*, 2018, 29, 1800987; *Adv. Funct. Mater.*, 2020, 30, 1908299).

Nevertheless, quantitatively measuring the temperature gradient after dissipating EM energy is still a challenge. On the other hand, EM wave-generated heat can be evaluated based on the following equation (Vinoy et al., Radar absorbing materials: from theory to design and characterization [B], Kluwer Academic Publishers):

$$\mathrm{W} = \mathrm{R} \cdot E^2 \cdot f \cdot \varepsilon_r \cdot \tan \delta e$$

where R refers to the size for specific material, E represents the power of electromagnetic waves, tan δ_e is the tangent loss and ε_r is the complex of permittivity.

To illustrate the connection between temperature gradient and wave harvesting, the microwave oven was employed to radiate the device (Figure R1a). The temperature difference between the background and the device was recorded at different radiation time. Note that the operation frequency of microwave oven (f = 2.5 GHz) ranges in our proposed frequency region.

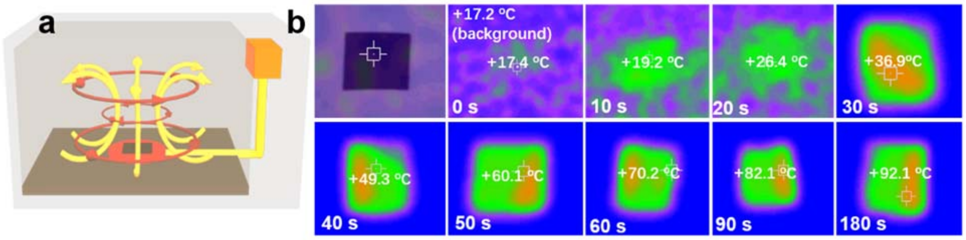

Figure R1 (Figure S19 in the revised supplementary materials) (a) Schematic illustration of measuring the changes of temperatures by microwave oven; (b) the changes of temperatures of device and background after radiating various temperatures. A4 paper was used as the background.

As shown in Figure R1b, taking the optimized EA-TE-3 device as a case, it can be clearly observed that the temperature-gradient between the device and background (A4 paper) gradually increases after rising the radiation time from 0 to 180 s.

We have added the above information in the revised manuscript and please see it in Note 6 (Supplementary material) and the second paragraph on Page 8 (revised manuscript).

Q3. The second concern is the EM wavelength. It is not clear what kind of wavelength was used for this research.

Response: In this work, the developed device exhibited an excellent EM-harvesting ability in the frequency region of 2~8 GHz, corresponding to a wavelength ranging from 15 to 3.75 cm. The investigation on the frequency region of 2~8 GHz is due to the fact that the electronic devices such as Bluetooth (2.4 GHz), wireless (2.4, 3.6 and 5.6 GHz), the four- and five-generation electronics (3~6 GHz) concentrate in this band range.

Reviewer #3: The authors proposed the new energy harvester using an electromagnetic wave-heat-electricity energy conversion material. One of the interesting points is the high thermoelectric properties of the carbon with the Sn nano-particles (beta phase). The biological cell-like behavior the Sn@C through the

cycled annealing is also useful information for the nanotechnology researchers. The non-dimensional figure of merit of the flexible composite, 0.72, is really high and interesting, as well as keeping high electromagnetic absorptance.

Response: We greatly appreciate Reviewer # 3 ’s highly positive comments.

Q1. The difference between the prepared 3 samples (EA-TE-1, EA-TE-2, EA-TE-3) is just annealing times in my understanding. Then, the most number of the smallest Sn nano-particles is made in EA-TE-3. It might be clear that the thermal conductivity is decreased when the phonons scattering centers are increased in the composite. The molecular dynamics discussion looks isolated. The authors calculated the phonons density of states as shown in Figs.5(d), (e), and they discuss thermal conductivity decrease shown in Fig.5 (f) using the values of phonon coupling. However, we cannot know the quality of carbon in EA-TE-1, 2, 3. It is difficult to understand the thermal conductivity change from the viewpoints of the phonon coupling values.

Response: We greatly appreciate Reviewer #3’s highly positive comments. We agree with the reviewer that an increase in the number of Sn nanoparticles leads to a reduction of the lattice thermal conductivity of the composite due to the increase in phonon scattering centers.

In the previous supplementary material, we investigated the effect of the number of nanoparticles on the lattice thermal conductivity of the composite via MD simulation. With the increase of the number of nanoparticles, the lattice thermal conductivity of the composite with the crystalline carbon matrix decreases dramatically (Figure S20d).

In addition to the number of the nanoparticles, the graphitization of the carbon matrix also plays a significant role in the lattice thermal conductivity of the composite. For the amorphous carbon matrix, the lattice thermal conductivity of the composite shows a little dependence on the number of nanoparticles (Figure S20e). Such distinct dependences of the lattice thermal conductivity could be understood by investigating the phonon coupling (characterized by the spectra overlap) between the nanoparticle and the carbon matrix (Figures 5d and 5e). A smaller phonon spectrum overlap for the composite with the amorphous carbon matrix indicates weaker phonon scattering at the interfaces and thus less reduction in the lattice thermal conductivity.

The XANES spectra (Figure 5c), Raman spectra (Figure S20) and X-ray photoelectron spectra (Figure S21) results demonstrated an improved graphitization level for the composite EA-TE-3. As a result, both the increase in the number of nanoparticles and enhanced phonon scattering at the nanoparticle-carbon matrix interface may lead to the lowest lattice thermal conductivity for EA-TE-3.

To closely link the MD simulation results with the experimental measurements, we have modified our discussion on the simulation results and please see the Note 7 in revised Supplementary material.

Note 7 graphitized degree analysis of carbon matrix

“As shown in Raman spectra (Figure S21 and S22), the I_D/I_G ratios are estimated to be 0.98, 0.67 and 0.55 for the 1st, 2nd and 3rd Sn@C samples, indicating the improved graphitized degree. This result is consistent with X-ray photoelectron energy spectra (XPS) result which 3rd Sn@C reaches the maximum surface areas of graphitized C-C bonds. According to the simulation, a higher graphitized degree of carbon matrix

would greatly benefit to the phonon coupling between Sn and C. As a result, it leads to an ultralow lattice thermal conductivity."

Reviewers' comments:

Reviewer #1 (Remarks to the Author):

In the revised version, the authors used a microwave to heat the sample and then measured the sample temperature and background temperature. However, it is not convincing for EM harvesting since the major innovation of this manuscript is to investigate the EM-electricity harvester. Particularly, the only property related to EM harvesting is Figure 4(e). However, authors only listed the ZT at different temperatures and there is no convincing evidence for harvesting EM. For example, we can list the ZT of any material in this Figure but it does not indicate EM harvesting. In addition, the detail of the Seebeck coefficient measurement is missing while the Seebeck coefficient is extremely critical for the thermoelectric behavior. Actually, the whole work is focused on fabrication and characterization of thermoelectric materials, instead of harvesting of EM. Based on these concerns, the review does not recommend acceptance of this manuscript.

Reviewer #3 (Remarks to the Author):

I understand the discussion on ultra-low thermal conductivity.

Let me remind authors that the maximum ZT will be $(S^2)/L$ if the Wiedemann-Franz law is valid. Here, S is the Seebeck coefficient and L is the Lorenz number.

In the present study, 0.73 will be the estimated maximum ZT(under the assumption of lattice thermal conductivity equals to zero) for EA-TE3 with -134 micro V/K. Please add the thermal conductivity measurement explanations in the Section of Methods. It might be very important to consider the ultra-low thermal conductivity from the viewpoint of measurements although the thermal conductivity reduction mechanisms are well explained.

Response Letter

Reviewer #1: In the revised version, the authors used a microwave to heat the sample and then measured the sample temperature and background temperature. However, it is not convincing for EM harvesting since the major innovation of this manuscript is to investigate the EM-electricity harvester. Particularly, the only property related to EM harvesting is Figure 4(e). However, authors only listed the ZT at different temperatures and there is no convincing evidence for harvesting EM. For example, we can list the ZT of any material in this Figure, but it does not indicate EM harvesting. In addition, the detail of the Seebeck coefficient measurement is missing while the Seebeck coefficient is extremely critical for the thermoelectric behavior. Actually, the whole work is focused on fabrication and characterization of thermoelectric materials, instead of harvesting of EM. Based on these concerns, the review does not recommend acceptance of this manuscript.

Response: We sincerely appreciate Reviewer #1's comments. We notice that the primary concern of Reviewer #1 is the EM harvesting issue, which is still a significant challenge in the scientific community. Fortunately, in the last months, we conducted considerable experiments and made a great progress on this issue.

The utilization of unwanted EM wave to generate electricity involves two procedures: i) dissipating ambient EM wave to heat via dielectric dissipation; ii) converting wasted heat to electricity based on thermoelectric effect. The dissipation ability can be evaluated according to the following equation (Vinoy et al., Radar absorbing materials: from theory to design and characterization [B], Kluwer Academic Publishers):

$$\mathrm{W} = D \cdot E^2 \cdot f \cdot \eta \qquad (1)$$

where D is a coefficient associated with the volume of an absorber, E and f represent the power and frequency of EM wave, respectively. η is EM dissipation factor, which can be calculated by the following equation (Lv, et al., *Adv. Mater*., 2018, 30, 1706343):

$$\eta = \varepsilon_r \cdot \tan\delta_E = \varepsilon_r \cdot \varepsilon'' / \varepsilon' \qquad (2)$$

where ε_r is the relative complex permittivity and $\tan\delta_E$ is the dielectric tangent loss, equaling to the ratio of $\varepsilon''/\varepsilon'$ (ε' and ε'' are real and imaginary part of permittivity values, respectively).

To maximize the utilization efficiency of EM energy, the η and ZT values of the as-synthesized Sn@C composites should be as large as possible. The 3rd Sn@C sample not only exhibited a strong EM dissipation factor over 16.6 ranging from 2.0 to 8.0 GHz (mainly working frequency for intelligent electronics such as 4/5 G wireless, WIFI, and Bluetooth) but also could achieve a ZT value of 0.62 at 473 K, meaning a possibility of coupled EM wave-thermal-electricity.

To provide an evidence for EM harvesting, we re-designed EM device. 4-legs of the 3rd Sn@C composite film and the flexible polydimethylsiloxane (PDMS) as an insulated substrate were employed to construct a device (Fig. R1). Before construction, a parylene-c (PL) layer with 0.5 cm in length was selectively deposited onto the front side of the Sn@C leg via a chemical vapor deposition (CVD) while Au-rectangle patterns were thermal-evaporated onto its back side using a specific mask. The selectively deposited PL layer is conducive to improving the EM dissipation factor and creating a large temperature gradient.

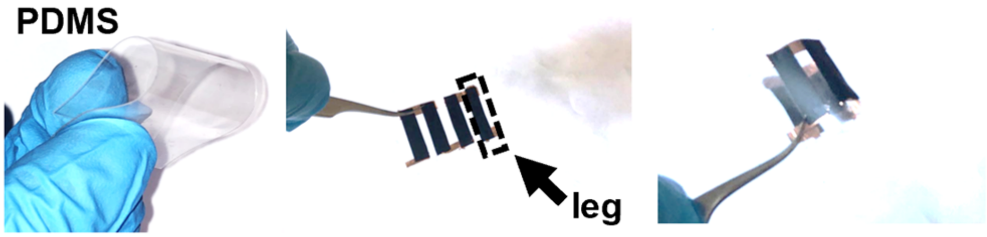

Fig. R1 Photograph of the PMDS substrate and the as-fabricated EM-electricity device.

The temperature difference (ΔT) was realized by EM radiating the Sn@C leg for 0~180 seconds using a homemade microwave emitting cavity. The magnetron (~100 W) as the core part of microwave emitting cavity could generate a ~2.45 GHz of EM waves (Fig. R2).

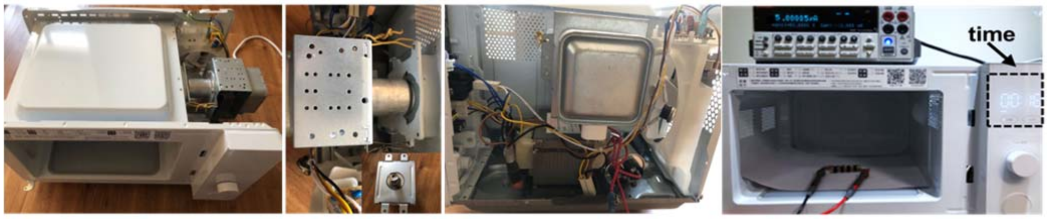

Fig. R2 Homemade microwave cavity and apparatus for the performance measurement. Note that the magnetron of microwave oven has a larger power (exceeding 1000 W) and sealed condition is requested to conduct microwave oven. In our case, we have changed the electronic circuit which the EM captivity can be used in an open stage. Meanwhile, the original magnetron (~1200 W) was replaced to a new one with a lower power (~100 W).

The ΔT as a function of radiation times was recorded via an infrared thermometer. The maximum ΔT could reach 36 K after radiating 150 seconds (Fig. R3). The open-circuit voltage (U_{oc}) output power and current-voltage of EM-electricity harvester were shown in Fig. R4. The measured U_{oc} exhibited a remarkable enhancement as radiation time increasing to 90 seconds and then gradually maintained a constant value (Fig. R4a). The maximum U_{oc} value reached ~14.3 mV after radiating 150 seconds, which was ascribed to the maximum ΔT .

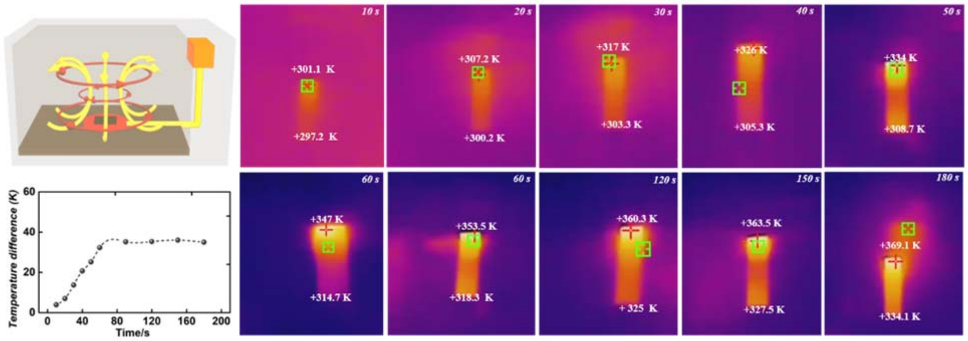

Fig. R3 Time dependent of temperature difference for the EM-electricity device after microwave radiating for 0~180 s.

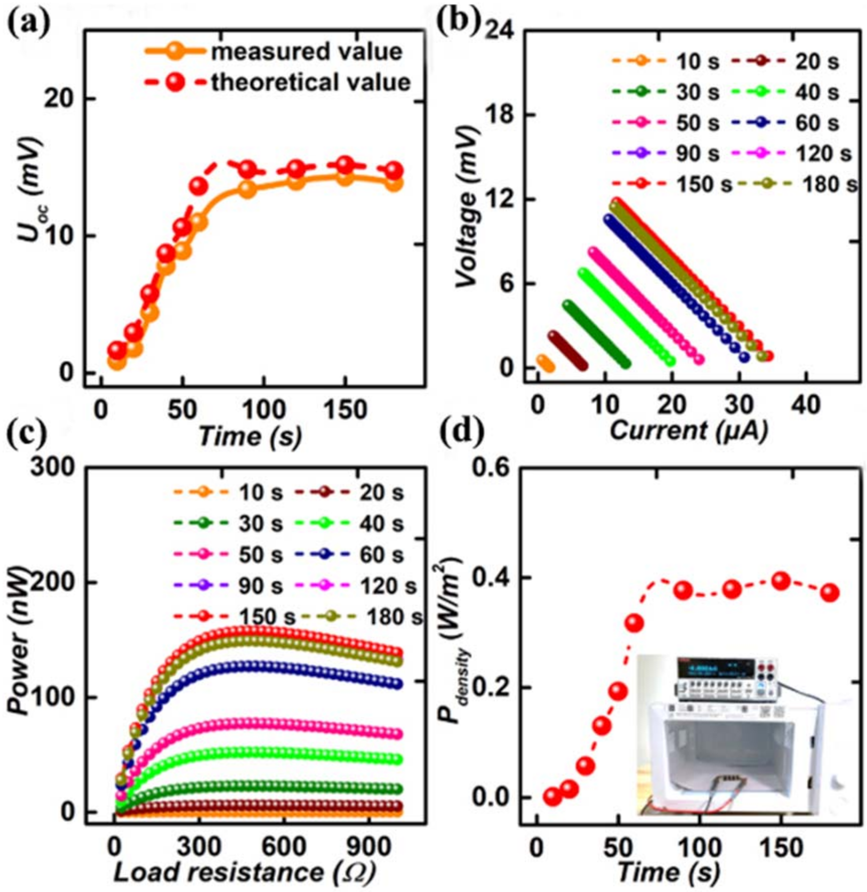

Fig. R4 (a) Time dependent of open-circuit (b) output voltage-current, (c) output power and (d) power density for the EM wave-electricity harvester. Note that the changed voltage and current could be recorded by alerting the load resistance from 0~1000 Ω.

Commonly, the theoretical U_{oc} value could be estimated by the equation $U_{oc}=\Delta T\times N\times\left|\overline{S}\right|$, where N is the number of leg, $\left|\overline{S}\right|$ is the average absolute Seebeck coefficient, ~105.4 μV/K. The measured U_{oc} values exhibited slightly lower than that of theoretical value, which was due to fact that the time delay (~15 seconds) from completing radiation to voltage-characterization would narrow the ΔT value. Fig. R4b plotted the output current versus voltage curves for the EM device after radiating various times and altered load resistance ranging from 0~1000 Ω. We observed that the maximum output voltage was approximately ~12 mV. Based on U-I curve, the output power (P_{out}) could be calculated by the follow expressions (Lu et al., *Energy Environ. Sci.*, 2020, 13, 1240):

$$P_{out}=I^2R_{ex}=(\frac{U_{oc}}{R_{in}+R_{ex}})^2R_{ex} \quad (4)$$

$$V_{out}=I\cdot R_{ex} \quad (5)$$

where I represents the output current, R_{ex} and R_{in} are the load resistance and internal resistance of leg, respectively, V_{out} refers to the output voltage. When the R_{ex} equals to R_{in}, P_{out} reached a maximum value. As plotted in Fig. R4c, a projected maximum P_{out} of ~157 nW could be achieved when radiating the EM device for 150 seconds and tuning the load resistance to 675 Ω. Apart from output power, the power density could be determined by $P_{density}=\frac{P_{max}}{N\cdot A}$, where A presents the crossed area of the leg. Similarly, after 150 seconds of

radiation, the EM device reached the maximum power density of 0.394 W/m^2 (Fig. R4c and R4d).

We have added the above discussion in the revised manuscript. Please see new Fig. 5, Fig. S20, S24 and S25.

Reviewer #3: I understand the discussion on ultra-low thermal conductivity. Let me remind authors that the maximum ZT will be (S^2)/L if the Wiedemann-Franz law is valid. Here, S is the Seebeck coefficient and L is the Lorenz number.

In the present study, 0.73 will be the estimated maximum ZT (under the assumption of lattice thermal conductivity equals to zero) for EA-TE3 with -134 micro V/K. Please add the thermal conductivity measurement explanations in the Section of Methods. It might be very important to consider the ultra-low thermal conductivity from the viewpoint of measurements although the thermal conductivity reduction mechanisms are well explained.

Response: Thank you very much for Reviewer #3's kind suggestion. We have added the thermal conductivity measurement explanations in the Section Methods and please see it in "Characterization of TE-related parameters" in Supplementary Materials.

REVIEWERS' COMMENTS

Reviewer #1 (Remarks to the Author):

The revised version is convincing and it is ready to publish.

Response to referees' letter

Reviewer #1: The revised version is convincing, and it is ready to publish.

Response: We sincerely appreciate Reviewer #1's support.
